# Thinning apples with 1-aminocyclopropane carboxylic acid: influence of cultivar, rate and timing

**DOI:** 10.3389/fpls.2026.1788111

**Published:** 2026-04-23

**Authors:** John A. Cline, Catherine J. Bakker

**Affiliations:** Ontario Crops Research Centre – Simcoe, Department of Plant Agriculture, Ontario Agricultural College, University of Guelph, Simcoe, ON, Canada

**Keywords:** abscission, chemical thinning, crop density, fruitlet drop, *Malus x domestica* L. Borkh

## Abstract

Crop load management of apple trees is necessary to improve fruit size, quality of apples and reduce biennial bearing. As an alternative to hand thinning, a new commercial thinning compound, 1-aminocyclopropane-carboxylic acid (ACC), has been in development for over a decade. A series of eight experiments were conducted to evaluate the efficacy of 1-aminocyclopropane- carboxylic acid (ACC) as a full bloom (FB) or post-bloom thinner for Ambrosia, Gala, Honeycrisp, and Crimson Crisp apple trees. Trees were treated with ACC at rates from 200-400 mg.L^-1^; different timings from FB to 20 mm; single, combination or sequential sprays of ACC with 6-benzyl adenine (6-BA) or 1-naphthaleneacetic acid (NAA); and two ACC formulations. Treatments were compared with untreated trees, hand thinned control (HTC), and grower control sprays of carbaryl, NAA, 6-BA, or combinations thereof. In all studies, single applications of ACC up to 400 mg.L^-1^ for fruitlet sprays and 600 mg.L^-1^ for full-bloom sprays failed to reduce fruit set or crop load compared to untreated trees. However, regression analyses indicated a significant linear or quadratic reduction in fruit set and crop load with increasing rates of ACC applied post-bloom in three of the six experiments. Ethylene emission peaked in fruits 1d after ACC was applied at 8-10 mm but was not detected when applied after 15-20 mm. Trees treated with carbaryl control sprays had comparable crop loads as the HTC and effective as a fruitlet thinner, but not in all experiments. ACC was not a sufficient substitute for carbaryl when used alone or tank mixed with 6-BA or used as a sequential spray. Further studies evaluating higher rates of ACC, using ACC in combination or sequence with other thinning agents, as well as understanding the reasons for its inconsistent thinning response are warranted.

## 1 Introduction

Gala, Honeycrisp, and Ambrosia apple trees are within the five most widely planted apple cultivars in Ontario making up 19%, 15% and 11% of the total Ontario acreage, respectively (Ontario Apple Growers, 2024). New plantings of these three cultivars are being established at a greater rate than other cultivars grown in Ontario, primarily due to their high returns and consumer appeal. However, horticulturally they all have unique production challenges. High quality 100–125 count size fruit (~155–190 g) often generate the highest returns, which requires fruit to be aggressively thinned to achieve this size. The goal of successful thinning is to reduce crop load and enhance fruit size, but obtaining a target size of 100–125 is difficult, especially for Ambrosia and Gala. Thinning early is necessary to reduce the high labour costs of thinning by hand in June after natural fruit abscission.

There is concern over the use of agricultural chemicals, particularly with respect to worker exposure and human health. Carbaryl (CB), a carbamate insecticide, is widely used to thin apples, and because of its low cost and effectiveness, particularly when combined with other thinners, it is often preferred over 6-benzyl adenine (6-BA) and 1-naphthaleneacetic acid (NAA). However, carbaryl is toxic to bees and other pollinating insects, and for environmental and health concerns, it is no longer used in Europe and has become increasingly restricted for use on apples in Canada (Health Canada, 2024). As recently as 2016, re-evaluation of carbaryl by the PMRA (Health Canada, 2016) led to further restricted use for thinning. This change led to a reduction from two to one applications per growing season with greater re-entry intervals after application. It is very possible that carbaryl may be prohibited in the coming years. Even if carbaryl remains registered, retailers may opt to no longer purchase fruit treated with it. This would have adverse consequences for apple growers in Canada, given their current limited thinning options. Growers in Ontario have identified the need for successful and predictable thinning strategies that do not involve Carbaryl.

Managing the crop load of apples remains a significant challenge to producers, in part because of the unpredictability of fruit set and fruitlet abscission. When left unchecked, apple trees often flower heavily and bear more fruit than necessary for a commercial crop and optimal returns (Solomakhin and Blanke, 2010). Because flower initiation in apple occurs the year prior to bloom, excessive crop load can lead to reduced bearing the follow year.

Chemical thinning remains the primary method used to reduce crop load. However, despite decades of research, it remains an unacceptably unpredictable part of apple production with large variation from year to year and between cultivars. Approaches for managing apple crop load include thinning at bloom and during early fruitlet development when fruit are between 5 mm (petal fall; PF) and 25 mm diameter, and hand thinning after natural abscission (June drop’). In general, there is low sensitivity to chemical thinners when fruitlets are small at PF (~4 mm diameter) followed by high sensitivity when fruitlets are between 8–15 mm, and low sensitivity once fruitlets reach ~20 mm (Wallis et al., 2022). The reason for this differing sensitivity is not fully understood, but weather (primarily temperature and solar radiation), cultivar, tree age, flower and initial fruit set, and carbohydrate status of the tree and developing fruit all play a role (Lakso, 2011). Growers typically cannot obtain ideal crop loads with just one chemical application, so a common strategy is to apply one or more thinners initiated at bloom, followed by PF and/or fruitlet applications before 15 mm when they become less responsive. In north America, carbaryl in combination with 6-BA or NAA is often a preferred treatment when moderate to heavy thinning is required.

Research over the past decade has indicated that the ethylene precursor 1-aminocyclopropane-1-carboxylic acid (ACC) is effective on Gold Rush (McArtney, 2011), Gale Gala and Golden Delicious (McArtney and Obermiller, 2012; Schupp et al., 2012; Beyá-Marshall et al., 2025) Fuji (Beyá-Marshall et al., 2025; Zanrosso et al., 2025) apple and also is efficacious for thinning peaches (Cline et al., 2020; McArtney et al., 2022) and pears (Cline et al., 2017).

In the context of Ontario’s growing regions, the aim of this study was to investigate the thinning efficacy of ACC as a post-bloom thinner for Ambrosia, Gala, Honeycrisp, and Crimson Crisp apple trees. In particular, different rates and timings with respect to fruit development (ranging from full bloom to 20 mm fruit diameter) were investigated and compared with untreated trees and industry standard sprays of Carbaryl, NAA, 6-BA, or combinations thereof.

## 2 Materials and methods

### 2.1 Methods common to all experiments

All experiments were conducted at the University of Guelph, Horticultural Experiment Station, Simcoe, ON (42°51’40” N, 80°16’8” W). All trees were trickle-irrigated daily during the growing season with an equivalent of ~2.5 cm of water weekly (adjusted for natural rainfall) on a schedule of 6 irrigation run-times per day every 4 h (20 min per event). Irrigation was performed using 2 L h^-1^ pressure-compensating emitters spaced 45 cm apart. Standard cultural and pest management practices for Ontario were followed (Ontario Ministry of Agriculture, Food, and Rural Affairs, 2018). Weeds were controlled within a 1-m strip on each side of the tree row using 1% (v/v) glyphosate applications made mid-May, June and July. A permanent sod culture was established at the time of planting in the row middle using a mixture of 40% perennial rye and 60% red fescue (Vineland Growers, Vineland, ON). The orchard soils consisted predominately of a Wattford fine sandy loam (Experiment 1, 2), Oakland loamy fine sand (Experiment 3), Wilsonville sandy loam (Experiment 4, 5) (Brunisolic Grey Brown Luvisol) (Presant and Acton, 1984) with good drainage and soil textures consisting mainly of glaciolacustrine over fine sand and loamy fine sand at depths greater than 50 cm (Hohner and Presant, 1989).

Treatments were applied using a commercial airblast sprayer (Model Turbomist 30P, Slimline Manufacturing, Penticton, BC) with spray volumes (L ha^-1^) for each experiment determined using a tree row volume (TRV) calculation (Sutton and Unrath, 1988). The TRV is based on in-row and between-row spacing, tree height and width, and for these studies, volumes were calculated to fully wet the tree canopy to near runoff (dilute basis). The sprayer tower boom was equipped with 12 air-induction nozzles (TRX80-VK, TeeJet Technologies, Louisville, KY) per boom (side). Depending on ambient wind conditions, the axial fan was at times turned off to prevent spray drift to the adjacent row.

Daily maximum and minimum air temperatures, precipitation and solar radiation were recorded by a weather station located within 500 m of the research orchards. Tree trunk circumference at 30 cm above the graft union was measured at the beginning and end of each growing season, from which trunk cross-sectional area (TCSA) was calculated. Return bloom was estimated by counting the number of flowering and non-flowering spurs on four primary limbs with fruit bud per tree. These limbs originated from the primary leader. At harvest, the total yield and number of fruit per tree were recorded. Mean fruit weight was calculated as the total yield divided by the total number of fruit per tree. Percent marketable yield was calculated as the yield of fruit that met minimum colour and diameter (60 mm) measurements divided by the total yield. Colour was determined qualitatively while harvest based on a minimum of 40% percentage of fruit surface covered with red. Crop load was calculated as the total number of fruit per tree at harvest divided by the TCSA in orchard to account for potential difference in canopy size. Municipal ground water was used as the source water for all spray mixtures.

In Experiments 1 and 2, ethylene evolution was measured in 2018 from five detached Gala and Honeycrisp fruitlets from untreated trees and select treated trees (modified from Cortens and Cline, 2019; McArtney, 2002). Fruitlets were removed, 24 h, 48 h, and 7 d after treatment. Fruitlets were weighed and placed immediately in a 125-mL glass jar with silicone septum inserted in the lid. Jars were sealed for ~4 h, the time required for sufficient ethylene to be measured in the sample. Five mL of headspace gas from the jar (of which 1 mL was used for the gas chromatograph (GC) column and the rest used to flush the system) was injected into a GC (Model 8610C, SRI Instruments, Torrance, USA) equipped with a 0.5 mL sample loop. The GC was configured to measure ethylene using a capillary column at 30-32°C (15 m x 0.32 mm Restek Rt-SPLOTTM, Chromatographic Specialties Inc., Brockville, ON, Canada) and flame ionisation detector. Pure ethylene (1 ppm) was used as the standard (BOG Gases, Mississauga, ON, Canada). Ethylene levels were expressed as parts per billion and converted to nanoliter ethylene per gram sample per hour.

### 2.2 Experiment 1 – Royal Gala/B.9

Experiments were conducted in 2017 and 2018 on a mature block of Royal Gala on B.9 planted in 2002 at a spacing of 2.5 m x 4.5 m (889 trees ha^-1^) and trained to a vertical axe system. The same research orchard was used in both years of the experiment. Trees were selected based on uniformity of bloom and tree growth, and any trees used in 2017 were re-randomised prior to assigning treatments in 2018.

Treatment sprays were applied to single tree plots, travelling at 3.1 km hr^-1^, 1380 kPa, 1212 L ha^-1^ (92 imp. gal acre^-1^). In addition, to minimise treatment interference caused by spray drift, experimental units were separated by at least one guard tree. The experimental design comprised a random complete block with seven replications and nine treatments. Treatments consisted of: I) an untreated control, II) a hand thinned control (HTC), III) a tank mix of 1500 mg L^-1^ CB (Tessenderlo Kerley Inc., Phoenix, Arizona USA) and 75 mg L^-1^ 6-BA (Maxcel, Valent Canada, Guelph, ON, Canada), IV) 200 mg L^-1^ ACC (10% (wt/wt) liquid formulation) Valent Biosciences, Libertyville, Illinois, USA), V) 300 mg L^-1^ ACC, VI) 400 mg L^-1^ ACC, VII) 75 mg L^-1^ 6-BA followed by 400 mg L^-1^ ACC, VIII) a tank mix of 200 mg L^-1^ ACC and 150 mg L^-1^ 6-BA, and IX) a tank mix of 300 mg L^-1^ ACC and 150 mg L^-1^ 6-BA. Treatment III) is considered a grower standard used for chemical fruitlet thinning Gala in our production region (Ontario Ministry of Agriculture, Food and Agribusiness, 2024). All the treatments applied on 1 June 2017 and 30 May 2018. Actual fruitlet diameter at the time of the aoplication was: king = 12.7 mm, n = 26; lateral = 11.5 mm, n = 26 in 2017 and king = 8.7 mm, n = 50; lateral = 7.3 mm, n = 50 in 2018. The follow up application of ACC for treatment vii) was applied on 7 June 2017 and 8 June 2018, when actual fruitlet diameters were: king = 17.3 mm, n = 26; lateral = 16.0 mm, n = 26 in 2017, and king = 18.1 mm, n = 50; lateral = 15.7 mm, n = 20 in 2018. All spray treatments included 0.125% LI 700 non-ionic spray adjuvant (Loveland Products Canada Inc., Dorchester, ON, Canada). After natural fruit drop, the HTC was thinned on 20 June 2017 and 21 June 2018 by singling fruit and spacing them approximately 10 cm apart. All other treatments were not hand thinned after natural fruit abscission to discern treatment effects on final crop load.

#### 2.2.1 Horticultural measurements

Four scaffold branches, two on the east and two on the west side of the tree, between 1 to 2.5 m above the ground, were selected prior to bloom to determine fruit set. On 11 May 2017 and 17 May 2018, the number of flower clusters per branch and the number of fruit per cluster were counted on each marked limb. The number of fruit set per limb were counted again on 7–8 Sept. 2017 and 21 June 2018. These data were averaged and used to calculate percent fruit set (number of fruit set divided by number of flowers).

Fruit were harvested on 18–20 Sept. 2017 and 18–20 Sept. 2018, and the total yield and number of fruit per tree recorded. A sample of approximately 100 fruit (1 bushel) per tree was placed in cold storage (~2°C) for subsequent grading on a commercial colour sorting grading line on 12, 15 Jan. 2018 and 5 Mar. 2019. All fruit from each sample were graded using a commercial sorting line (MAF RODA, MAF Agrobotic, Montauban Cedex, France) which relied on cameras and sensors to weigh and size each individual fruit. Individual fruit weights and sizes were recorded. Fruit were then separated according to their weight into 15 size categories expressed as an average count size category, which was the number of apples needed to fill a 20 kg box (Canadian Food Inspection Agency, 2011). The total weight of fruit per tree was calculated for each count size category: 1) < 96 g (count size 216), 2) 96–108 g (count size 198), 3) 109–116 g (count size 175), 4) 117–126 g (count size 163), 5) 127–137 g (count size 150), 6) 138–151 g (count size 138), 7) 152–168 g (count size 125), 8) 169–190 g (count size 113), 9) 191–215 g (count size 100), 10) 216–237 g (count size 88), 11) 238–264 g (count size 80), 12) 265–297 g (count size 72), 13) 298–339 g (count size 64), 14) 340–396 g (count size 56), and 15) ≥ 397 g (count size 48).

### 2.3 Experiment 2 – Honeycrisp/M.9 (2017), Honeycrisp/M.26 (2018)

Experiments were conducted in 2017 on a mature block of Honeycrisp on M.9 rootstock and in 2018 on a mature block of Honeycrisp on M.26. The Honeycrisp/M.9 trees were planted in 2012 at a spacing of 0.85 m x 3.5 m (3361 trees ha^-1^) and trained to a super spindle system. The Honeycrisp/M.26 trees used in 2018 were planted in 2008 at a spacing of 1.2 m x 4.0 m (2083 trees ha^-1^) and trained to a super spindle system.

Treatments were applied to single tree plots, travelling at 3.2 km hr^-1^, 1380 kPa, 1246 L ha^-1^ (112 imp. gal acre^-1^). In addition, to minimise treatment interference caused by spray drift, experimental units were separated by at least one guard tree. The experimental design comprised a randomised complete block with seven replications and ten treatments. Treatments consisted of: I) an untreated control, II) a hand-thinned control, III) 1500 mg L^-1^ CB (Sevin XLR Plus, Bayer CropScience Inc, Calgary, AB, Canada), IV) 10 mg L^-1^ NAA (Fruitone L, AMVAC Chemical Corporation, Los Angeles, CA, U.S.A), V) a tank mix of 1500 mg L^-1^ CB and 10 mg L^-1^ NAA, VI) 200 mg L^-1^ ACC (10% (wt/wt) liquid formulation), VII) 300 mg L^-1^ ACC, VII-IX) 400 mg L^-1^ ACC, and X) 10 mg L^-1^ NAA followed by 400 mg L^-1^ ACC. Treatments iii-viii) and the NAA application for treatment X) were applied on 1 June 2017 and 30 May 2018 targeting a fruitlet diameter of 8–10 mm. To avoid the production of excessively large Honeycrisp at harvest, thinning treatments excluded the use of the cytokinin 6-BA (which can increase cell division and fruit size) and relied on NAA and CB; NAA is the preferred fruitlet thinner for Honeycrisp grown in the Great Lakes region since, unlike 6-BA, it will not stimulate cell division and can reduce fruit size compared to other chemical thinners (Wallis et al., 2022). While a concern in cultivars like Gala and Delicious, the development of pygmy fruit has not been observed when NAA has been used as a fruitlet thinner for Honeycrisp. Actual fruitlet diameter at the time of the application was: king = 13.4 mm, n = 26; lateral = 12.0 mm, n = 26 in 2017 and king = 9.2 mm, n = 50; lateral = 6.7 mm, n = 50 in 2018. Treatment ix) and the follow up application of ACC for treatment x) were applied on 7 June 2017 and 8 June 2018 targeting a fruitlet diameter of 15–20 mm. Actual fruitlet diameter at the time of the follow up application was: king = 18.4 mm, n = 26; lateral = 17.4 mm, n = 26 in 2017 and king = 19.0 mm, n = 50; lateral = 16.6 mm, n = 50 in 2018. All spray treatments included 0.125% LI 700 non-ionic spray adjuvant (Loveland Products Canada Inc., Dorchester, ON, Canada). After natural fruit drop, the HTC was thinned on 20 June 2017 and 18 June 2018 by singling fruit and spacing them approximately 10 cm apart. All other treatments were not hand thinned after natural fruit abscission to discern treatment effects on final crop load.

#### 2.3.1 Horticultural measurements

Four scaffold branches, two on the east and two on the west side of the tree, between 1 to 2.5 m above the ground, were selected prior to bloom to determine fruit set. On 11 May 2017 and 18 May 2018, the number of flower clusters per branch and the number of fruit per cluster were counted on each marked limb. The number of fruit set per limb were counted again on 8 September 2017 and 18 June 2018. These data were averaged and used to calculate percent fruit set (number of fruit set divided by number of flowers).

Fruit were harvested on 12 Sept. 2017 and 24 Sept. 2018, and the total yield and number of fruit per tree recorded. A sample of approximately 100 fruit (1 bushel) was placed in cold storage (~2°C) for subsequent grading on a commercial colour sorting grading line on 18 Jan. 2018 and 27 Feb. 2019 as described for Experiment 1.

### 2.4 Experiment 3 – Ambrosia/B.9

Experiments were conducted in 2017 and 2018 on a mature block of Ambrosia on B.9 planted in 2005 at a spacing of 2.0 m x 4.5 m (1111 trees ha^-1^) and trained to a vertical axe system.

Treatments were applied to single tree plots, travelling at 3.2 km hr^-1^, 1380 kPa, 969 L ha^-1^. In addition, to minimise treatment interference caused by spray drift, experimental units were separated by at least one guard tree. The experimental design comprised a randomised complete block with seven replications and ten treatments. Treatments consisted of: I) a hand-thinned control, II) 1500 mg L^-1^ CB (Sevin XLR Plus), III) 150 mg L^-1^ 6-BA (Maxcel, Valent Canada, Guelph, ON, Canada), IV) a tank mix of 1500 mg CB and 75 mg/L 6-BA, V) 200 mg L^-1^ ACC (10% (wt/wt) liquid formulation), VI) 300 mg L^-1^ ACC, VII-VIII) 400 mg L^-1^ ACC, IX) 75 mg L^-1^ 6-BA followed by 400 mg L^-1^ ACC, X) a tank mix of 200 mg L^-1^ ACC and 150 mg L^-1^ 6-BA, and XI) a tank mix of 300 mg L^-1^ ACC and 150 mg L^-1^ 6-BA. There were an insufficient number of trees to include an untreated control treatment for this experiment. Treatments II-VII), X), XI) and the application of 6-BA for treatment ix) were applied on 1 June 2017 and 30 May 2018 targeting a fruitlet diameter of 8–10 mm. Actual fruitlet diameter at the time of the 8–10 mm application was: king = 10.7 mm, n = 26; lateral = 8.7 mm, n = 26 in 2017 and king = 8.5 mm, n = 50; lateral = 6.6 mm, n = 50 in 2018. Treatment VIII) and the follow up application of ACC were applied on 7 June 2017 and 5 June 2018 targeting a fruitlet diameter of 15–20 mm. Actual fruitlet diameter was king = 14.6 mm, n = 26; lateral = 12.6 mm, n = 26 in 2017 and king = 13.8 mm, n = 50; lateral = 11.6 mm, n = 50 mm in 2018. All spray treatments included 0.125% LI 700 non-ionic spray adjuvant (Loveland Products Canada Inc., Dorchester, ON, Canada). After natural fruit drop, the HTC was thinned on 20 June 2017 and 19 June 2018 by singling fruit and spacing them approximately 10 cm apart.

#### 2.4.1 Horticultural measurements

Two scaffold branches in 2017 (one on the east and one on the west side of the tree) and four scaffold branches in 2018 (two on the east and two on the west side of the tree) between 1 to 2.5 m above the ground, were selected prior to bloom to determine fruit set. On 10 May 2017 and 22 May 2018, the number of flower clusters per branch and the number of fruit per cluster were counted on each marked limb. The number of fruit set per limb were counted again on 22 Sept. 2017 and 19 June 2018. These data were averaged and used to calculate percent fruit set (number of fruit set divided by number of flowers).

Fruit were harvested on 28 Sept. 2017 and 4 Oct. 2018, and the total yield and number of fruit per tree recorded. A sample of approximately 100 fruit (1 bushel) was placed in cold storage (~2°C) for subsequent grading on a commercial colour sorting grading line on 15, 17 Jan. 2017 and 27 Feb., 5 Mar. 2019 as described for Experiment 1.

### 2.5 Experiment 4 – Ambrosia/M.9

Experiments were conducted in 2021 on a mature block of Ambrosia on M.9 rootstock planted in 2012 at a spacing of 0.9 m x 4 m (2,778 trees ha^-1^) and trained to a super spindle system. Treatments were applied to two-tree plots, travelling at 3.2 km hr^-1^, 1380 kPa, 816 L ha^-1^. In addition, to minimise treatment interference caused by spray drift, experimental units were separated by at least one guard tree. The experimental design comprised a randomised complete block with six replications and eight treatments. Treatments consisted of: I) untreated control; II) hand-thinned control; III-IV) a 10% wt/wt liquid formulation of ACC (Valent BioSciences Corp., Libertyville, IL) applied at 200 mg L^-1^ or 400 mg L^-1^ ACC at PF and 18–20 mm fruitlet diameter; V-VI) a granular formulation of ACC (40% wt/wt ACC; Accede SG™) applied at 200 mg L^-1^ or 400 mg L^-1^ ACC at PF and 18–20 mm fruitlet diameter; VII) 1000 mg L^-1^ CB (Sevin XLR, Tessenderlo Kerley Inc., Phoenix, AZ) tank mixed with 75 mg L^-1^ 6-BA (MaxCel; Valent BioSciences Corp., Libertyville, IL) applied at 12–15 mm fruitlet diameter; and VIII) 1000 mg L^-1^ CB tank mixed with 75 mg L^-1^ 6-BA applied at 12–15 mm fruitlet diameter followed by 400 mg L^-1^ ACC (Accede SG™)at 18–20 mm fruitlet size. All spray treatments included 0.125% (v/v) Agral 90 non-ionic surfactant containing 92% nonylphenoxy polyethoxy ethanol (Syngenta Canada, Guelph, ON).

ACC treatments were applied on the mornings of 25 May (PF) and 10 June 2021, which corresponded with king fruitlet diameter of 7.3 mm (± 2.1 mm; n = 50) and lateral fruitlet size of 6.5 mm (± 1.5 mm; n = 50) and on 10 June with a king fruitlet diameter of 20.3 mm (± 2.1 mm; n = 50) and lateral fruitlet size of 18.6 mm (± 2.9 mm; n = 50). The tank mix of CB and 6-BA treatments were applied once on the morning of 30 May 2021 which corresponded with a king fruitlet diameter of 10.4 mm (± 1.6 mm; n = 50) and lateral fruitlet diameter of 9.6 mm (± 1.2 mm; n = 50). The hand-thinned control trees were hand thinned on 26 June 2021 by removing all but one fruit per cluster and spacing fruit ~10 cm apart. All other treatments were not hand thinned after natural fruit abscission to discern treatment effects on final crop load. The date of full bloom of Ambrosia trees was 10 May 2021.

#### 2.5.1 Horticultural measurements

Four scaffold branches, two on the east and two on the west side of the tree, between 1 to 2.5 m above the ground, were selected prior to bloom to determine fruit set. On 5 May 2021, the number of flower clusters per branch and the number of fruit per cluster were counted on each marked limb. The number of fruit set per limb were counted again on 25 June 2021. These data were averaged and used to calculate percent fruit set (number of fruit set divided by number of flowers).

Fruit were harvested on 6 Oct. 2021, and the total yield and number of fruit per tree recorded. A sample of 60 fruit from each experimental unit t (30 per tree) was placed in cold storage (~2°C) for subsequent grading on a commercial grading line on 27 Nov. 2021 as described for Experiment 1.

### 2.6 Experiment 5 – Crimson Crisp/M.9

Experiments were conducted in 2022 on a mature block of Crimson Crisp on M.9 rootstock planted in 2012 at a spacing of 0.75 m x 3.5 m (3,617 trees ha^-1^) and trained to a super spindle. Treatments were applied to two-tree plots, travelling at 4 km hr^-1^, 1380 kPa, 379 L ha^-1^. In addition, to minimise treatment interference caused by spray drift, experimental units were separated by at least one guard tree. The experimental design comprised a randomised complete block with six replications and seven treatments. Treatments consisted of: I) untreated control; II) hand-thinned control; III-VI) ACC (10% (wt/wt) liquid formulation; Valent BioSciences Corp., Libertyville, IL) applied at 300, 400, 500, and 600 mg L^-1^ at full bloom, and; VII) 1500 mg L^-1^ CB (Sevin XLR, Tessenderlo Kerley Inc., Phoenix, AZ) tank mixed with 75 mg L^-1^ 6-BA (MaxCel; Valent BioSciences Corp., Libertyville, IL) applied at 12–15 mm fruitlet diameter. All spray treatments included 0.05% (v/v) Regulaid non-ionic surfactant containing (Kalo Inc., Overland Park, KS).

ACC treatments were applied on the mornings of 18 May (full bloom; FB). The tank mix of CB and 6-BA treatments were applied once on the morning of 2 June 2022 which corresponded with a king fruitlet diameter of 12.4 mm (± 1.5 mm; n = 50) and lateral fruitlet diameter of 10.3 mm (± 1.1 mm; n = 50). The hand-thinned control trees were hand thinned on 24 June 2022 by removing all but one fruit per cluster and spacing fruit ~10 cm apart. All other treatments were not hand thinned after natural fruit abscission to discern treatment effects on final crop load.

#### 2.6.1 Horticultural measurements

Four scaffold branches, two on the east and two on the west side of the tree, between 1 to 2.5 m above the ground, were selected prior to bloom to determine fruit set. On 11 May 2022, the number of flower clusters per branch and the number of fruit per cluster were counted on each marked limb. The number of fruit set per limb were counted again on 15 June 2022. These data were averaged and used to calculate percent fruit set (number of fruit set divided by number of flowers).

Fruit were harvested on 5 Oct. 2022, and the total yield and number of fruit per tree recorded. A sample of 60 fruit from each plot (30 per tree) was placed in cold storage (~2°C) for subsequent grading on a commercial grading line on 27 Nov. 2022 as described for Experiment 1.

### 2.7 Statistical analyses

Data were subjected to analysis of variance using the PROC GLIMMIX procedure of SAS (SAS 9.4, SAS Institute, Cary, NC). Mean fruit weight was adjusted by using crop load as a co-variate. Mean separation using Tukey’s HSD was used to separate treatment means (*P ≤* 0.05). A single degree of freedom orthogonal comparison was performed to evaluate the rate effects of ACC. Shapiro-Wilk test was used to test the normality of residuals. Scatterplots of studentised residuals were visually observed to test the assumption that errors were not heterogeneous. In cases where there were large deviations from assumptions, data were transformed using log-, or square root, or arcsine square root transformation prior to analysis. All graphs were created using SigmaPlot version 13.0 (Systat Software, Inc., Germany).

## 3 Results

### 3.1 Experiment 1 – Royal Gala/B.9

There were significant treatment effects on fruit set of Royal Gala apple in 2017 (*P* = 0.0030); however, there were few differences between thinning treatments with ACC compared to the untreated and HTC or the grower standard treatment (1500 mg L^-1^ CB) tank mixed with 75 mg L^-1^ 6-BA) (**Table 1**). The only exception was the tank mix of 200 mg L^-1^ ACC plus 150 mg L^-1^ 6-BA which resulted in significantly higher fruit set compared to the HTC. The rate of ACC did not influence fruit set based on orthogonal contrasts. Based on the means separation, most of the ACC treatments performed similarly to the grower standard treatment and HTC despite large numerical differences.

In 2018, there was a highly significant (*P* = 0.0009) treatment effect on the number of flowering spurs with zero fruit; however, there were few statistically significant differences among the treatments based on the means separation (**Table 1**). Only trees sprayed with the grower standard CB tank mixed with 6-BA had significantly more flower spurs with zero fruit compared to the untreated control. The rate of ACC did not influence the number of flower spurs with 0, 1, 2, or 4 fruit. CB tank mixed with 6-BA treatment resulted in fewer spurs with one fruit compared to the HTC. There was a strong treatment effect (*P* < 0.0001) on the number of spurs with two fruit, but ACC applied alone was similar to the untreated control. Only applications CB tank mixed with 6-BA and ACC treatments that also included 6-BA were statistically similar to the HTC. A similar trend was observed in the number of spurs with three fruit. ACC applied alone was similar to the untreated control; however, 300 mg L^-1^ ACC and the ACC treatments that included 6-BA were statistically similar to the HTC and grower standard treatments. The orthogonal contrasts indicted that the number of spurs with three fruit responded in a cubic fashion with increasing rates of ACC. There was no treatment effect on the number of spurs with four or five fruit, although orthogonal contrasts indicated a curvilinear response with increasing rates of ACC in the number of spurs with five fruit. In 2017 and 2018, there was no treatment effect on the percent of flowering spurs with fruit the following spring.

**Table 1:** Influence of various rates and combinations of carbaryl (CB), 1-aminocyclopropane-1-carboxylic acid (ACC) and 6-benzyladenine (6-BA) on fruit set in 2017 and 2018 of Royal Gala/B.9 apples planted in 2002.

	Application timing/fruitlet diameter^a^	Fruit set (no. fruit per 100 flower clusters)	Percentage of flower spurs with indicated number of fruit											Return bloom (% of spurs with flowers)^b^	
Treatment (mg L^-1^)				0		1		2		3		4	5		
2017															
Untreated control	–	117.8	ab	–		–		–		–		–	–	0.94	(64.7)
Hand-thinned control	June drop	57.8	b	–		–		–		–		–	–	1.02	(72.2)
1500 CB + 75 6-BA	8-10 mm	72.3	b	–		–		–		–		–	–	1.00	(70.9)
200 ACC	8-10 mm	130.4	ab	–		–		–		–		–	–	0.99	(69.6)
300 ACC	8-10 mm	98.7	ab	–		–		–		–		–	–	0.90	(61.7)
400 ACC	8-10 mm	107.4	ab	–		–		–		–		–	–	1.12	(81.2)
75 6-BA/400 ACC	8-10/15-20 mm	92.8	ab	–		–		–		–		–	–	0.99	(70.0)
200 ACC + 150 6-BA	8-10 mm	156.4	a	–		–		–		–		–	–	0.91	(61.9)
300 ACC + 150 6-BA	8-10 mm	93.8	ab	–		–		–		–		–	–	1.06	(76.3)
*P*		0.0030		–		–		–		–		–	–	0.1864	
Rate of ACC		NS		–		–		–		–		–	–	NS	
2018															
Untreated control	–	116.7		28.6	b	40.2	ab	21.8	a	6.6	abc	1.9	0.9	58.1	
Hand-thinned control	June drop	48.7		52.0	ab	48.0	a	0.0	c	0.0	d	0.0	0.0	64.3	
1500 CB + 75 6-BA	8-10 mm	37.0		68.1	a	26.5	b	5.0	c	0.4	cd	0.0	0.0	68.6	
200 ACC	8-10 mm	107.3		36.5	b	31.3	ab	23.1	a	8.0	a	1.1	0.0	68.3	
300 ACC	8-10 mm	96.3		34.2	b	39.0	ab	23.4	a	3.3	abcd	0.2	0.0	62.6	
400 ACC	8-10 mm	101.7		38.4	b	32.9	ab	19.7	ab	7.0	ab	1.8	0.3	60.8	
75 6-BA/400 ACC	8-10/15-20 mm	58.8		55.8	ab	32.3	ab	9.4	bc	2.2	abcd	0.3	0.0	52.3	
200 ACC + 150 6-BA	8-10 mm	121.6		52.2	ab	34.6	ab	11.4	abc	1.7	abcd	0.0	0.0	42.6	
300 ACC + 150 6-BA	8-10 mm	69.2		49.0	ab	37.0	ab	11.8	abc	1.4	bcd	0.7	0.1	59.9	
*P*		0.1367		0.0009		0.0181		<0.0001		0.0002		0.2541	0.2753	0.3124	
Rate of ACC		NS		NS		NS		NS		C*		NS	Q*	NS	

Mean values with the same letter within a given column are not significantly different according to Tukey's HSD test at*P≤* 0.05. NS, *, **, indicates not significant, and significant differences at *P* = 0.05 and *P* = 0.01, respectively. Q and C refer to quadratic and cubic relationships.

^a^ Treatment application dates were as follows: 8-10 mm (1 June 2017, 30 May 2018), 15-20 mm (7 June 2017, 8 June 2018). In 2017, the 1500 CB + 75 6-BA treatment was applied on 30 May.

^b^ Data from 2017 were transformed using an arcsine square root transformation prior to analyis. Values in brackets are mean values back-transformed to the original scale.

Thinning treatments applied in 2017 did not affect any yield parameters (**Table 2**). In 2018, thinning treatments had a significant effect on the measured yield parameters; however, ACC applied alone, regardless of rate, generally had similar total yield, total number of fruit per tree, marketable yield and adjusted mean fruit weight as the untreated and HTC. Only the treatments that incorporated 6-BA resulted in a statistically similar total yield or total fruit number as the grower standard CB tank mixed with 6-BA treatment. Treatments that resulted in a greater degree of thinning generally resulted in higher percent marketable yield and mean fruit weight although there were few significant differences among the treatments as indicated by means separation.

**Table 2:** Influence of various rates and combinations of carbaryl (CB), 1-aminocyclopropane-1-carboxylic acid (ACC) and 6-benzyladenine (6-BA) on tree growth and yield in 2017 and 2018 of Royal Gala/B.9 apples planted in 2002.

Treatment (mg L^-1^)	Application timing/fruitlet diameter^a^	Total fruit yield (kg per tree)	Total number of fruit (no. per tree)		Percent marketable yield (%)		Adjusted mean fruit weight (g)		Crop load (no. fruit per TCSA)^b^		
2017											
Untreated control	–	25.1		194		53.0		138		2.1	
Hand-thinned control	June drop	19.0		134		56.2		147		1.1	
1500 CB + 75 6-BA	8-10 mm	18.3		126		61.9		150		1.6	
200 ACC	8-10 mm	33.9		259		55.0		139		2.4	
300 ACC	8-10 mm	33.3		255		53.0		133		2.1	
400 ACC	8-10 mm	30.2		230		55.1		131		1.9	
75 6-BA/400 ACC	8-10/15-20 mm	31.0		239		47.6		136		2.1	
200 ACC + 150 6-BA	8-10 mm	31.3		245		51.9		142		3.2	
300 ACC + 150 6-BA	8-10 mm	22.4		170		57.9		139		1.7	
*P*		0.0719		0.0520		0.9581		0.4358		0.3323	
Rate of ACC		NS		NS		NS		NS		NS	
2018											
Untreated control	–	57.0	a	394	a	55.3	ab	146	ab	2.8	ab
Hand-thinned control	June drop	33.6	abc	200	cd	71.4	a	165	a	1.3	b
1500 CB + 75 6-BA	8-10 mm	23.3	c	140	d	72.1	a	163	a	1.3	b
200 ACC	8-10 mm	54.1	a	385	ab	46.6	ab	146	ab	2.8	ab
300 ACC	8-10 mm	45.1	abc	350	abc	41.4	b	133	b	3.2	ab
400 ACC	8-10 mm	52.0	ab	403	a	42.0	b	135	b	4.0	a
75 6-BA/400 ACC	8-10/15-20 mm	35.4	abc	221	bcd	57.8	ab	157	ab	1.7	ab
200 ACC + 150 6-BA	8-10 mm	29.1	bc	185	cd	66.2	ab	158	ab	1.9	ab
300 ACC + 150 6-BA	8-10 mm	33.1	abc	223	bcd	60.6	ab	156	ab	2.6	ab
*P*		0.0001		<0.0001		0.0007		0.0037		0.0263	
Rate of ACC		NS		NS		NS		NS		NS	

Mean values with the same letter within a given column are not significantly different according to Tukey's HSD test at *P≤* 0.05. NS, *, **, indicates not significant, and significant differences at *P* = 0.05 and *P* = 0.01, respectively. Q and C refer to quadratic and cubic relationships.

^b^ Determined by dividing the total number of fruit harvested with the TCSA measured in fall.

Thinning treatments applied in 2017 did not have a significant effect on the distribution of fruit in the count size categories (**Table 3**). In 2018, thinning treatments had a significant effect on the amount of fruit in the 125 (*P* = 0.0042), 138 (*P* < 0.0001), 150 (*P* = 0.0019) and 163 (*P* = 0.0011) size categories. CB tank mixed with 6-BA reduced the amount of fruit in the 125 and 138 size categories compared to the untreated control. All other thinning treatments resulted in a similar number of fruit in the 138-size category to the HTC except for the 400 mg L^-1^ rate of ACC. The HTC, grower standard thinning treatment, and 400 mg L^-1^ ACC resulted in the smallest amount of fruit in the 150-size category, which were all significantly lower than the untreated control but similar to the other chemical thinning treatments. There were few significant differences in the number of fruit in the 163-size category among the treatments and all thinning treatments were similar to the HTC and grower standard control except for the 300 mg L^-1^ rate of ACC. The amount of fruit in the 163-count size category exhibited a complex cubic relationship in response to increasing rates of ACC (*P* = 0.05).

**Table 3:** Influence of various rates and combinations of carbaryl (CB), 1-aminocyclopropane-1-carboxylic acid (ACC) and 6-benzyladenine (6-BA) on the weight of fruit per count size in 2017 and 2018 of Royal Gala/B.9 apples planted in 2002.

	Application timing/fruitlet diameter^a^	Amount of fruit per tree in each size category (kg)^b^															
Treatment (mg L^-1^)		72	80	88	100	113	125		138		150		163		175	198	216
2017																	
Untreated control	–	0.03	0.1	0.4	1.1	3.5	4.1		6.8		3.3		3.4		1.8	0.8	0.0
Hand-thinned control	June drop	0.00	0.0	0.1	1.7	3.9	4.5		4.5		2.1		0.9		0.6	0.5	0.1
1500 CB + 75 6-BA	8-10 mm	0.02	0.2	0.5	1.6	4.1	4.6		3.9		2.3		0.7		0.2	0.0	0.0
200 ACC	8-10 mm	0.00	0.1	0.5	1.9	5.2	6.5		7.6		4.7		4.0		1.9	1.2	0.3
300 ACC	8-10 mm	0.00	0.0	0.1	1.3	4.2	5.3		9.3		6.1		4.4		1.6	0.8	0.2
400 ACC	8-10 mm	0.00	0.0	0.1	0.9	4.4	6.4		6.9		4.6		2.6		1.2	1.9	1.0
75 6-BA/400 ACC	8-10/15-20 mm	0.01	0.0	0.3	1.2	3.9	5.6		8.6		6.1		3.3		1.2	0.7	0.2
200 ACC + 150 6-BA	8-10 mm	0.00	0.1	0.3	1.6	5.5	6.4		6.6		3.4		2.4		0.7	1.4	2.1
300 ACC + 150 6-BA	8-10 mm	0.00	0.0	0.2	0.9	3.6	3.6		4.4		2.4		2.6		2.7	1.6	0.3
*P*		0.4996	0.6646	0.7326	0.9084	0.7413	0.5293		0.2413		0.0531	0.0942			0.5153	0.7093	0.6505
Rate of ACC		NS	NS	NS	NS	NS	NS		NS		NS		NS		NS	NS	NS
2017																	
Untreated control	–	0.0	0.0	0.4	5.1	10.0	13.2	a	11.3	ab	7.8	a	4.3	ab	2.1	2.2	0.5
Hand-thinned control	June drop	0.0	0.5	2.3	6.5	9.3	6.1	ab	4.7	bc	2.1	b	1.2	b	0.3	0.3	0.2
1500 CB + 75 6-BA	8-10 mm	0.0	0.7	1.6	5.0	6.8	4.3	b	2.6	c	1.2	b	0.5	b	0.2	0.1	0.2
200 ACC	8-10 mm	0.0	0.0	0.8	5.7	12.4	13.1	a	10.6	ab	5.5	ab	2.8	ab	1.8	1.1	0.4
300 ACC	8-10 mm	0.0	0.0	0.2	2.0	7.1	8.6	ab	9.4	ab	6.3	ab	5.8	a	2.2	2.4	1.1
400 ACC	8-10 mm	0.0	0.0	0.2	3.9	10.5	9.7	ab	11.9	a	5.4	ab	4.4	ab	2.4	2.8	0.7
75 6-BA/400 ACC	8-10/15-20 mm	0.0	0.2	0.7	6.1	9.9	9.3	ab	5.6	abc	2.0	b	0.8	b	0.6	0.0	0.1
200 ACC + 150 6-BA	8-10 mm	0.0	0.0	0.7	3.5	8.0	6.5	ab	4.8	bc	3.0	ab	1.4	b	0.7	0.2	0.2
300 ACC + 150 6-BA	8-10 mm	0.0	0.3	1.4	4.0	5.8	5.9	ab	6.3	abc	4.0	ab	2.4	ab	1.4	1.2	0.5
*P*		–	0.2395	0.0643	0.4828	0.6631	0.0042		<0.0001		0.0019	0.0011			0.1119	0.0506	0.3001
Rate of ACC		–	NS	NS	NS	NS	NS		NS		NS		C*		NS	NS	NS

Mean values with the same letter within a given column are not significantly different according to Tukey's HSD test at *P≤* 0.05. NS, *, **, indicates not significant, and significant differences at *P* = 0.05 and *P* = 0.01, respectively. Q and C refer to quadratic and cubic relationships.

^a^ Treatment application dates were as follows: 8-10 mm (1 June 2017, 30 May 2018), 15-20 mm (7 June 2017, 8 June 2018). In 2017, the 1500 CB + 75 6-BA treatment was applied on 30 May.

^b^ Fruit diameter equivalents for each count size: 72 = 89-92 mm, 80 = 84.5-89 mm, 88 = 83-84.5 mm, 100 = 79-83 mm, 113 = 76-79 mm, 125 = 73-76 mm, 138 = 70-73 mm, 150 = 67-70 mm, 163 = 64-67 mm, 175 = 60-64 mm, 198 = 57-60 mm, 216 = <57 mm.

**Figure 1:**
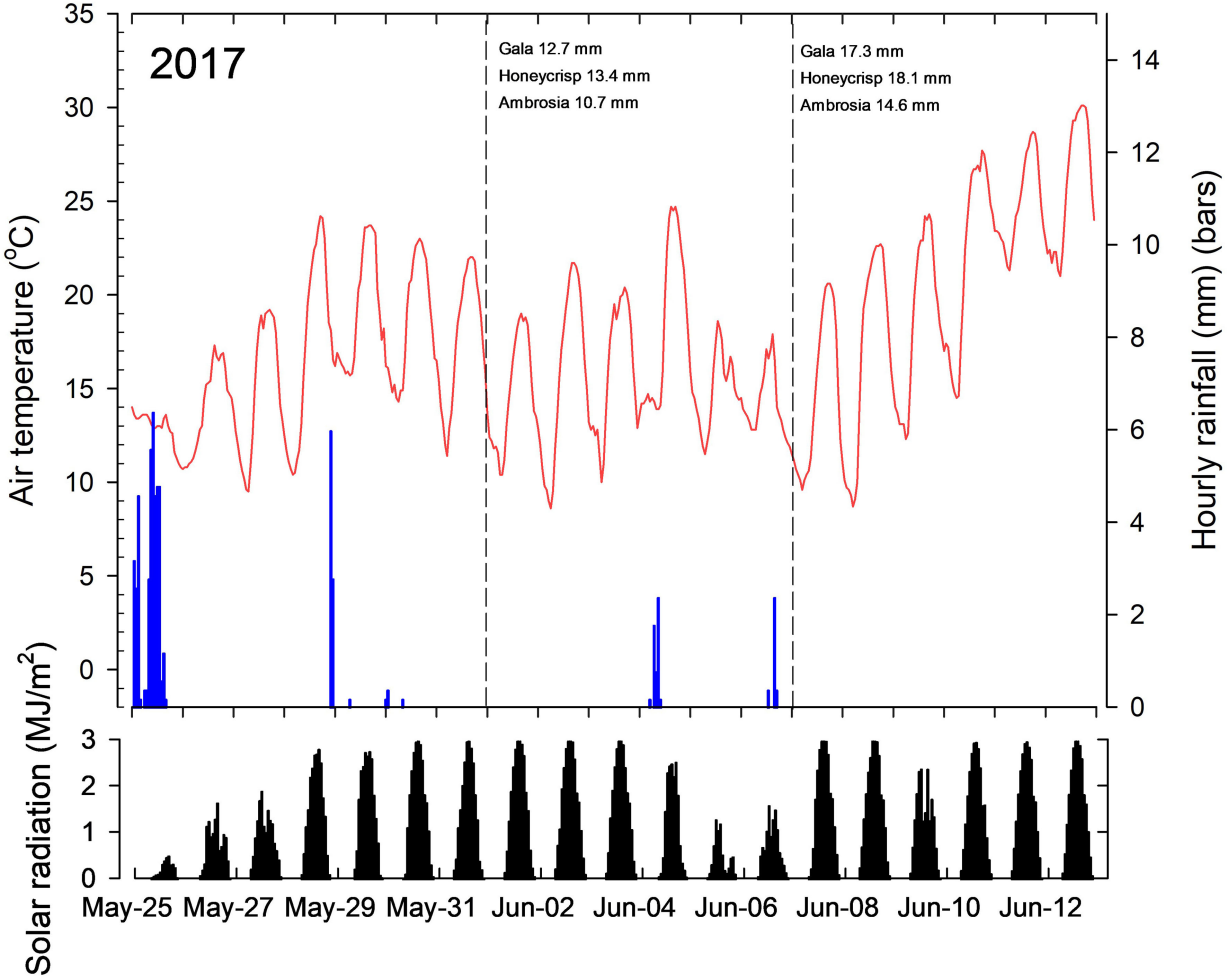
Average hourly air temperatures (red line), total hourly precipitation (blue bars) and solar radiation (black bars) seven days prior to the first application of treatments, and seven days after the last treatments applications to Gala (Expt 1), Honeycrisp (Expt 2) and Ambrosia (Expt 3) trees in 2017. The dashed vertical line in the top panel indicates the day the treatments were applied and the average diameter of king fruitlets measured on that day. Data recorded at a research weather station located within 500 m of the research orchards.

**Figure 2:**
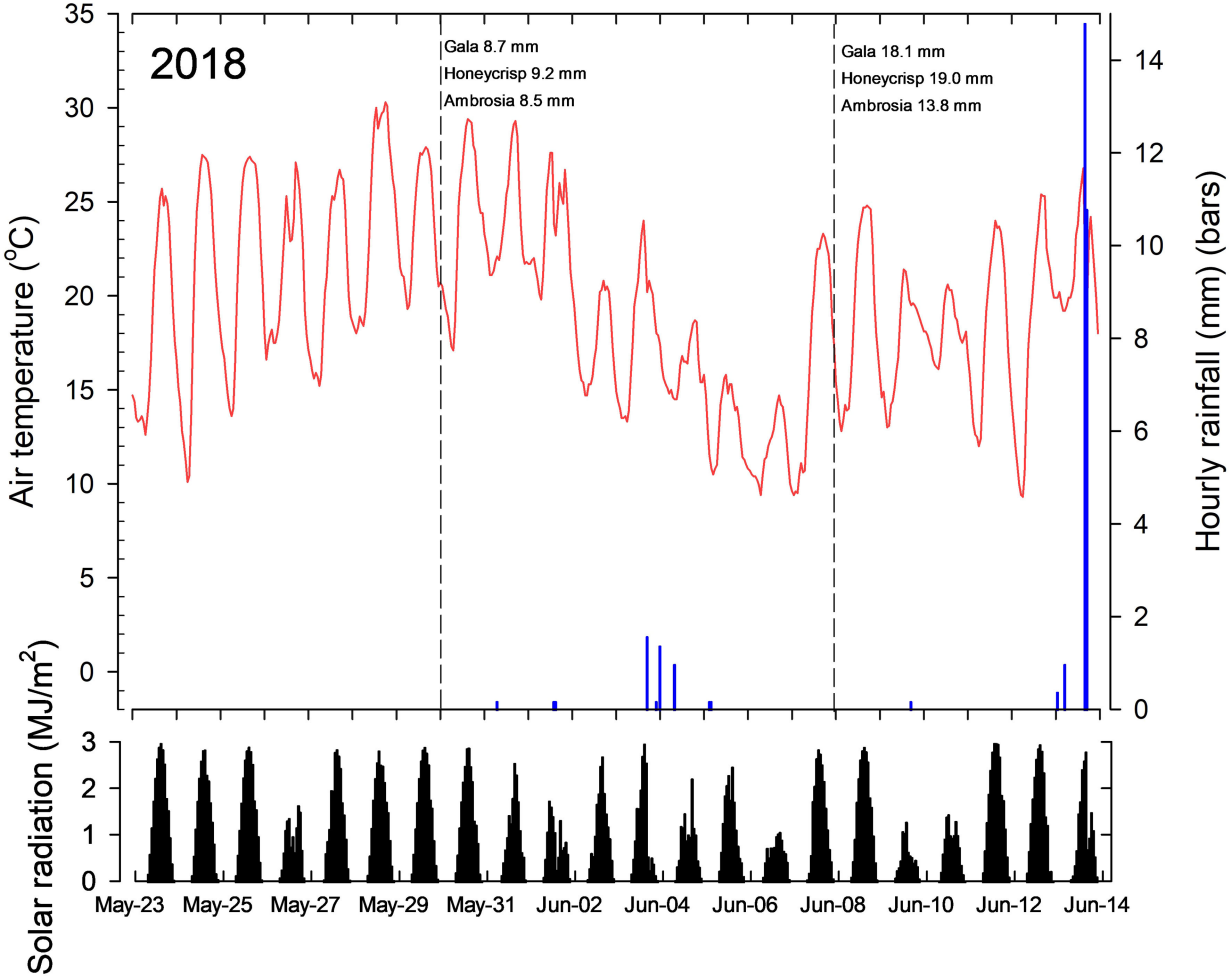
Average hourly air temperatures (red line), total hourly precipitation (blue bars) and solar radiation (black bars) seven days prior to the first application of treatments, and seven days after the last treatments applications to Gala (Expt 1), Honeycrisp (Expt 2) and Ambrosia (Expt 3) trees in 2018. The dashed vertical line in the top panel indicates the day the treatments were applied and the average diameter of king fruitlets measured on that day. Data recorded at a research weather station located within 500 m of the research orchards.

Air temperatures one week prior to the application of the first treatments on 1-Jun-2017 were relatively warm ranging from 10 to 25°C with high solar radiation levels four day prior to treatment application (**Figure 1**). Similar conditions prevailed seven days after with high solar radiation. Air temperatures increased daily after the second applications were made on 7-June with high solar radiation levels. Precipitation levels over this period was low, except for 25-May. In 2018, air temperatures were more variable following the first application of treatments on 30-May (**Figure 2**). Air temperatures approached 30°C for the first three days when treatments were made, but declined thereafter with daily highs between 15°C and 20°C, with decrease solar radiation levels. Air temperatures were more moderate following the second application of treatments on 8-June with fluctuating solar radiation levels.

Applications of ACC increased ethylene evolution from fruitlets 24 h after application compared to all other treatments (*P* = 0.0004) (**Table 4**). The rise in ethylene was transient and not detected 7 d after application. No ethylene was detected in fruitlets 24 hr and 7 d after the secondary application of 400 mg L^-1^ ACC on 7 June when fruit were 15–20 mm or with fruit from the untreated control and the 75 mg L^-1^ 6-BA tank mixed with 400 mg L^-1^ ACC treatments.

**Table 4:** Influence of various rates and combinations of carbaryl (CB), 1-aminocyclopropane-1-carboxylic acid (ACC) and 6-benzyladenine (6-BA) on the rate of ethylene evolution in dettached fruitlets in 2017 of Royal Gala/B.9 apples planted in 2002.

	Application timing/fruitlet diameter^a^	Rate of ethylene evolution (nl Ethylene g fruit^-1^ hr^-1^)				
Treatment (mg L^-1^)		24 hours after first spray	7 days after first spray		24 hours after second spray	7 days after second spray
Untreated control	–	0.91	b	0.04	0.00	0.00
Hand-thinned control	June drop	–		–	–	–
1500 CB + 75 6-BA	8-10 mm	0.68	b	0.00	–	–
200 ACC	8-10 mm	–		0.04	–	–
300 ACC	8-10 mm	10.83	a	0.07	–	–
400 ACC	8-10 mm	17.00	a	0.13	–	–
75 6-BA/400 ACC	8-10/15-20 mm	1.73	b	0.07	0.00	0.00
200 ACC + 150 6-BA	8-10 mm	–		–	–	–
300 ACC + 150 6-BA	8-10 mm	–		–	–	–
*P*		0.0004		0.5569	–	–
Rate of ACC		–		NS	–	–

Mean values with the same letter within a given column are not significantly different according to Tukey's HSD test at P≤ 0.05. NS indicates not significant at P = 0.05.

^a^ Treatment application dates were as follows: 8-10 mm (1 June 2017), 15-20 mm (7 June 2017). The 1500 CB + 75 6-BA treatment was applied on 30 May.

### 3.2 Experiment 2 – Honeycrisp/M.9 (2017), Honeycrisp/M.26 (2018)

Thinning treatments had a significant effect on fruit set in the 2018 Honeycrisp experiment (*P* < 0.0001) but not in 2017 (*P* = 0.0740, **Table 5**). In 2018, all thinning treatments significantly reduced fruit set compared to the untreated control except all ACC treatments applied alone. Applications of the grower standard 1500 mg L^-1^ CB, 300 or 400 mg L^-1^ ACC (8–10 mm fruitlet diameter) and 10 mg L^-1^ NAA applied alone or followed by 400 mg L^-1^ ACC resulted in a moderate reduction in fruit set that was similar to the HTC. The greatest treatment effect on fruit set was observed with a tank mix of CB and 10 mg L^-1^ NAA applied at 8–10 mm fruitlet diameter; however, fruit set for this treatment was similar to the grower standard CB, 10 mg L^-1^ NAA and the sequential spray of NAA and ACC, based on the means separation. Application of 400 mg L^-1^ ACC at 15–20 mm fruitlet diameter was ineffectual in thinning Honeycrisp, whereas when applied at 8–10 mm, it reduced fruit set by 25% compared to the untreated control. There was also no added benefit of the secondary spray of 400 mg L^-1^ ACC at 15–20 mm when combined with the primary spray of 10 mg L^-1^ NAA at 8–10 mm. With increasing rates of ACC, fruit set decreased in a linear (P = 0.05) and curvilinear fashion (*P* = 0.05) in 2017 and 2018, respectively.

**Table 5:** Influence of various rates, combinations and spray timings of carbaryl (CB), 1-naphthaleneacetic acid (NAA) and 1-aminocyclopropane-1-carboxylic acid (1-ACC) on fruit set in 2017 of Honeycrisp/M.9 planted in 2012 and in 2018 of Honeycrisp/M.26 apples planted in 2008.

	Application timing/fruitlet diameter^a^	Fruit set (no. fruit per 100 flower clusters)	Percentage of flower spurs with indicated number of fruit											Return bloom (% of spurs with flowers)^b^		
Treatment (mg L^-1^)				0		1		2		3		4	5			
2017																
Untreated control		120.9		–		–		–		–		–	–	0.78	ab	(49.5)
Hand-thinned control	June drop	66.5		–		–		–		–		–	–	1.00	ab	(70.6)
1500 CB	8-10 mm	68.0		–		–		–		–		–	–	0.82	ab	(53.4)
10 NAA	8-10 mm	80.1		–		–		–		–		–	–	0.80	ab	(51.0)
1500 CB + 10 NAA	8-10 mm	57.7		–		–		–		–		–	–	1.00	ab	(70.5)
200 1-ACC	8-10 mm	76.8		–		–		–		–		–	–	0.55	b	(26.9)
300 1-ACC	8-10 mm	79.9		–		–		–		–		–	–	0.53	b	(25.9)
400 1-ACC	8-10 mm	132.8		–		–		–		–		–	–	1.19	a	(86.3)
400 1-ACC	15-20 mm	107.8		–		–		–		–		–	–	0.90	ab	(61.0)
10 NAA/400 1-ACC	8-10/15-20 mm	105.1		–		–		–		–		–	–	0.91	ab	(62.6)
*P*		0.0740		–		–		–		–		–	–	0.0324		
Rate of 1-ACC		Q*		–		–		–		–		–	–	Q**		
2018																
Untreated control		73.0	ab	45.6	c	41.9	ab	10.0	abc	1.6	ab	0.4	0.5	9.7	b	
Hand-thinned control	June drop	48.1	bc	53.6	bc	46.4	a	0.0	e	0.0	b	0.0	0.0	15.6	b	
1500 CB	8-10 mm	38.9	cd	64.4	b	31.6	bc	4.0	bcde	0.0	b	0.0	0.0	30.7	b	
10 NAA	8-10 mm	33.9	cd	68.9	ab	28.3	cd	2.6	cde	0.3	b	0.0	0.0	27.7	b	
1500 CB + 10 NAA	8-10 mm	10.8	d	84.2	a	15.8	d	0.2	de	0.0	b	0.0	0.0	60.1	a	
200 1-ACC	8-10 mm	62.8	abc	52.2	bc	34.9	abc	11.3	ab	1.7	ab	0.0	0.0	7.2	b	
300 1-ACC	8-10 mm	46.7	bc	65.2	ab	26.7	cd	6.8	bcde	1.3	ab	0.0	0.0	28.4	b	
400 1-ACC	8-10 mm	54.4	bc	58.6	bc	30.6	bc	8.6	bcd	2.1	ab	0.1	0.0	31.7	b	
400 1-ACC	15-20 mm	85.9	a	41.6	c	36.6	abc	17.5	a	3.7	a	0.5	0.2	11.0	b	
10 NAA/400 1-ACC	8-10/15-20 mm	36.2	cd	69.4	ab	25.8	cd	3.8	bcde	0.5	b	0.4	0.0	14.2	b	
**Note:** Mean values with the same letter wi		<0.0001		<0.0001		<0.0001		<0.0001		0.0004		0.4401	0.1899	<0.0001		
Rate of 1-ACC		L*		L**		L***		NS		NS		NS	L**	L**		

Mean values with the same letter within a given column are not significantly different according to Tukey's HSD test at P≤ 0.05. TCSA, trunk cross-sectional area. NS, *,**, ***, indicates not significant, and significant differences at P = 0.05, P = 0.01, and P = 0.001 respectively. L, Q, C refers to linear, quadratic, and cubic relationships.

^a^ Treatment application dates were as follows: 8-10 mm (1 June 2017, 30 May 2018), 15-20 mm (7 June 2017, 8 June 2018).

^b^ Return bloom data from the 2017 trial were transformed using an arcsine square root transformation prior to analyis. Values in brackets are mean values back-transformed to the original scale.

The percentage of flowering spurs with zero, one, two or three fruit was significantly affected by the thinning treatments in 2018 (*P* < 0.0001, **Table 5**). Thinning treatments that included NAA, the grower standard CB, and 300 mg L^-1^ ACC resulted in significantly more flower spurs that set zero fruit than the untreated control. The HTC (which had not been thinned when fruit set was measured) was not significantly different from the grower standard CB treatment according to the means separation. The percentage of spurs with zero fruit on trees treated the combination of CB and NAA had significantly more spurs with zero fruit than the HTC treatment. In contrast, all the other treatments had similar percentage of spurs with zero fruit as the HTC. Contrasts indicated that increasing rates of ACC increased the percentage of spurs with zero fruit in a linear fashion (*P* = 0.01). Treatments that provided a moderate to high degree of thinning, namely those which included NAA and the 300 mg L^-1^ rate of ACC significantly reduced the percentage of spurs with one fruit compared to the untreated control. Increasing rates of ACC decreased the percentage of spurs with one fruit in a linear fashion (*P* = 0.001). The HTC and CB tank mixed with NAA reduced the percentage of spurs with two fruit compared to the untreated control. Treatments including CB or NAA and the 300 mg L^-1^ rate of ACC had similar percentages of spurs with two fruit. Treatments that resulted in a greater degree of thinning generally had the lowest percentage of spurs with three fruit, although there were few statistically significant differences among the treatments based on the means separation. Overall, data on the percentage of spurs with different numbers of fruit revealed that application of 400 mg L^-1^ ACC at 15–20 mm fruitlet diameter as a stand-alone treatment or following a spray of NAA was ineffective at reducing the number of fruit per flower cluster.

Thinning treatments applied in 2017 and 2018 had a significant effect on return bloom (*P* = 0.0324 and *P* < 0.0001, respectively; **Table 5**). Applications of 200 and 300 mg L^-1^ ACC in 2017 had the lowest return bloom, but it was similar to the UTC and the other treatments except the 400 mg L^-1^ ACC at 8–10 mm fruitlet treatment, which had the highest level of return bloom in the spring of 2018. Return bloom in spring 2019 reflected the degree of thinning observed in 2018 indicated by treatments that resulted in the lowest fruit set generally having a higher percentage of flowering spurs the following spring. The highest return bloom in 2019 was recorded on trees treated with a tank mix of CB and NAA. Return bloom in spring following thinning treatments applied in 2017 showed a quadratic response (*P* = 0.01) to increasing rates of ACC. In 2018, increasing rates of ACC increased return bloom in a positive linear response (*P* = 0.01). There was no difference in return bloom between the 400 mg L^-1^ ACC treatment applied at 8–10 mm versus 15–20 mm fruitlet size in either year. There was also no added benefit of the secondary spray of 400 mg L^-1^ ACC when combined with the primary spray of 10 mg L^-1^ NAA in either year of the experiment.

In 2018, total yield and number of fruit per tree were lower on trees that had reduced crop loads, such as the tank mix of CB and NAA (**Table 6**). The application of 400 mg L^-1^ ACC at 15–20 mm fruitlet diameter was an ineffective thinner and had similar crop load and mean fruit weight to the untreated control. For those treatments that reduced crop load, there was no benefit in improved fruit weight. There was also no added benefit, for any measured yield parameter, of the secondary spray of 400 mg L^-1^ ACC at 15–20 mm when combined with the primary spray of 10 mg L^-1^ NAA. For reference, total yields and number of fruit per tree for the HTC in 2018 were within target of commercial norms, but the grower control of the combined CB and NAA treatment was approximately 70% below the expected ~20 T/ha, indicating its level of overthinning.

**Table 6:** Influence of various rates, combinations and spray timings of carbaryl (CB), 1-naphthaleneacetic acid (NAA) and 1-aminocyclopropane-1-carboxylic acid (ACC) on tree growth and yield in 2017 of Honeycrisp/M.9 apples planted in 2012 and in 2018 of Honeycrisp/M.26 apples planted in 2008.

Treatment (mg L^-1^)	Application timing/fruitlet diameter^a^	Total fruit yield (kg per tree)	Total number of fruit (no. per tree)			Percent marketable yield (%)	Adjusted mean fruit weight (g)		Crop load (no. fruit per TCSA)^b^		
2017											
Untreated control		8.4		52	ab	76.4	a	163	ab^c^	6.7	ab
Hand-thinned control	June drop	9.6		43	b	94.6	a	226	a	5.1	b
1500 CB	8-10 mm	10.2		62	ab	89.3	a	173	ab	7.9	ab
10 NAA	8-10 mm	9.8		71	ab	84.6	a	154	ab	9.3	ab
1500 CB + 10 NAA	8-10 mm	6.7		44	b	94.7	a	148	b	5.9	b
200 ACC	8-10 mm	10.2		74	ab	88.2	a	164	ab	10.2	ab
300 ACC	8-10 mm	10.8		92	a	85.9	a	159	ab	12.5	a
400 ACC	8-10 mm	7.2		52	ab	79.2	a	135	b	7.1	ab
400 ACC	15-20 mm	8.9		57	ab	79.1	a	154	ab	7.8	ab
10 NAA/400 ACC	8-10/15-20 mm	8.4		56	ab	81.1	a	155	ab	8.0	ab
*P*		0.2717		0.0322		0.0230		0.0139		0.0042	
Rate of ACC		Q*		Q**		Q*		NS		Q**	
2018											
Untreated control		26.4	ab	130	ab	52.4	ab	228		4.8	abc
Hand-thinned control	June drop	18.7	b	86	bc	61.4	ab	211		3.1	bcd
1500 CB	8-10 mm	18.3	b	69	cd	62.9	ab	258		2.8	cde
10 NAA	8-10 mm	19.0	ab	76	c	68.2	a	235		2.5	de
1500 CB + 10 NAA	8-10 mm	7.6	c	25	d	72.6	a	244		0.8	e
200 ACC	8-10 mm	27.0	ab	135	ab	51.4	ab	237		5.1	ab
300 ACC	8-10 mm	20.9	ab	98	bc	57.5	ab	224		3.7	abcd
400 ACC	8-10 mm	19.8	ab	93	bc	61.5	ab	228		3.5	abcd
400 ACC	15-20 mm	28.7	a	153	a	43.8	b	225		5.4	a
10 NAA/400 ACC	8-10/15-20 mm	21.3	ab	93	bc	59.2	ab	242		4.0	abcd
*P*		<0.0001		<0.0001		<0.0001		0.1705		<0.0001	
**Note:** Mean values with the same letter wi		L*		L**		L**		NS		L*	

Mean values with the same letter within a given column are not significantly different according to Tukey's HSD test at *P≤* 0.05. TCSA, trunk cross-sectional area. NS, *, **, ***, indicates not significant, and significant differences at *P* = 0.05, *P* = 0.01, and *P* = 0.001 respectively. L, Q, C refers to linear, quadratic, and cubic relationships.

^a^ Treatment application dates were as follows: 8-10 mm (1 June 2017, 30 May 2018), 15-20 mm (7 June 2017, 8 June 2018).

^b^ Determined by dividing the total number of fruit harvested with the TCSA measured in fall.

^c^ The means separation does not reflect all significant comparisons. The following treatments are significantly different: Hand-thinned control and 10 NAA (8-10 mm)/400 ACC (15-20mm), Hand-thinned control and 400 ACC (15-20mm).

Total yield in 2017 was not significantly affected by the thinning treatments but significant treatment differences were observed in 2018 (*P* < 0.0001) (**Table 6**). In 2018, treatments with higher fruit set generally resulted in higher total fruit yield per tree. All ACC treatments and NAA applied alone at 8–10 mm fruitlet diameter resulted in a statistically similar total yield as both the untreated and HTCs based on the means separation. Total yield (*P* = 0.05) was reduced in a linear fashion with increasing rate of ACC.

Total number of fruit per tree was significantly affected by the thinning treatments in 2017 (*P* = 0.0322) and 2018 (*P* < 0.0001) (**Table 6**). The HTC and CB tank mixed with NAA had the fewest fruit in 2017 and a curvilinear reduction in total fruit number with increasing rates of ACC was also observed (*P* = 0.01). Total number of fruit per tree in 2018 followed similar trends as fruit set, with the 400 mg L^-1^ rate of ACC applied at 15–20 mm fruitlet diameter having the highest number of fruit, followed by 200 mg L^-1^ ACC, and the untreated control. Applications of 10 mg L^-1^ NAA with or without a secondary application of ACC, 300 mg L^-1^ ACC and 400 mg L^-1^ ACC applied at 8–10 mm fruitlet diameter resulted in a similar number of fruit as the HTC. NAA with or without a second spray of ACC, CB tank mixed with NAA, 300 mg L^-1^ and 400 mg L^-1^ ACC applied at 8–10 mm fruitlet diameter resulted in a similar number of fruit as the grower standard 1500 mg L^-1^ CB treatment. A significant negative linear response (*P* = 0.01) in fruit number to increasing rates of ACC was observed.

In 2017, a significant difference in percent marketable yield among treatments (*P* = 0.0230) and a significant curvilinear response to rates of ACC was observed (*P* = 0.05); however, Tukey’s HSD test indicated that all treatments were similar (**Table 6**). Percent marketable fruit in 2018 was significantly influenced by the thinning treatments (*P* < 0.0001). NAA without a follow up spray of ACC and CB tank mixed with NAA resulted in the highest percent of marketable fruit, while 400 mg L^-1^ACC applied at 15–20 mm fruitlet diameter resulted in the lowest percent of marketable fruit.

Increasing rates of ACC increased marketable percent in a linear fashion (*P* = 0.01).

Mean fruit weight, adjusted for crop load, was significantly affected by the thinning treatments in 2017 (P = 0.0139) but not in 2018. Mean fruit weight in 2017 was highest for the HTC and lowest for CB tank mixed with NAA and 400 mg L^-1^ ACC applied at 8–10 mm fruitlet diameter. Mean fruit weight was unaffected by the application rate of ACC in either year.

Thinning treatments had a significant effect on crop load in 2017 and 2018 (*P* = 0.0042 and *P* < 0.0001, respectively) which corresponded with the reduction in number of fruit per tree. Crop load for most treatments in 2017 exceeded six fruit per TCSA, which is approaching the maximum ideal crop load per tree for Honeycrisp, with the exception of the HTC and CB tank mixed spray with NAA. In 2017, crop load was highest on trees treated with 300 mg L^-1^ ACC at 8–10 mm fruitlet diameter and lowest for the HTC and CB tank mixed with NAA treatments; with the exception of 300 mg L^-1^ ACC at 8–10 mm fruitlet diameter, no treatment different from the tank mix of CB and NAA. In 2018, crop load was highest following application of 400 mg L^-1^ ACC at 15–20 mm fruitlet diameter, and lowest with CB tank mixed with NAA. Only CB tank mixed with NAA and NAA applied without a secondary application of ACC reduced crop loads compared to the untreated check. In 2017 and 2018, crop load was reduced in a curvilinear (*P* = 0.01) and linear fashion (*P* = 0.001), respectively (**Table 6**).

Thinning treatments had a significant effect on the size distribution of fruit in both years of the experiment (**Table 7**). In 2017, the amount of fruit in the medium to large 80 (*P* = 0.0010), 88 (*P* < 0.0001) and 100 (*P* = 0.0030) size categories, and the 163 (*P* = 0.0233), 175 (*P* < 0.0001), 198 (*P* = 0.0002), and 216 (*P* = 0.0017) small size categories were affected by the thinning treatments. In 2018, the large sizes 48 (*P* = 0.0010), 56 (*P* = 0.0003) 64 (*P* = 0.0057), and 80 (*P* = 0.0244), and moderate sizes 88 (*P* < 0.0001), 100 (*P* < 0.0001), 113 (*P* = 0.0002), and 125 (*P* = 0.0170) were affected by the thinning treatments. Generally, treatments that reduced crop load shifted fruit size to the higher size categories with fewer fruit in the small size categories. In 2017, there were significant quadratic and cubic relationships between application rate of ACC and the weight of fruit in the 113, and 163 to 216 size categories. In 2018, linear and quadratic relationships existed between rates of ACC and the weight of fruit in the 88 and 100 size categories, with increasing rates of ACC reducing the number of fruit in these categories.

**Table 7:** Influence of various rates, combinations and spray timings of carbaryl (CB), 1-naphthaleneacetic acid (NAA) and 1-aminocyclopropane-1-carboxylic acid (ACC) on the weight of fruit per count size in 2017 of Honeycrisp/M.9 apples planted in 2012 and in 2018 of Honeycrisp/M.26 apples planted in 2008.

	Application iming/fruitlet diameter^a^	Amount of fruit per tree in each size category (kg)^b^																										
Treatment (mg L^-1^)		48		56		64		72	80		88		100		113		125		138	150	163		175		198		216	
2017																												
Untreated control		0.0		0.0		0.0		0.1	0.5	ab	0.8	bc^c^	2.0	ab	2.3		1.7		1.2	0.3	0.2	a	0.3	b	0.3	b	0.2	ab^d^
Hand-thinned control	June drop	0.0		0.0		0.1		0.4	1.5	a	2.1	a	3.3	a	1.4		0.5		0.2	0.1	0.0	a	0.0	b	0.0	b	0.0	b
1500 CB	8-10 mm	0.0		0.0		0.2		0.4	1.0	ab	1.5	ab	2.3	ab	1.6		1.3		0.7	0.4	0.4	a	0.3	b	0.1	b	0.1	b
10 NAA	8-10 mm	0.0		0.0		0.1		0.1	0.3	b	0.4	bc	0.9	b	1.2		1.4		1.6	1.0	1.2	a	0.6	b	0.6	ab	0.2	ab
1500 CB + 10 NAA	8-10 mm	0.0		0.0		0.2		0.4	0.6	ab	0.7	bc	1.3	ab	1.4		1.0		0.6	0.2	0.1	a	0.0	b	0.1	b	0.1	b
200 ACC	8-10 mm	0.0		0.0		0.0		0.0	0.5	ab	0.4	bc	1.5	ab	1.4		1.2		1.3	0.7	1.1	a	0.9	ab	0.8	ab	0.4	ab
300 ACC	8-10 mm	0.0		0.0		0.0		0.1	0.1	b	0.2	c	0.6	b	0.9		0.8		1.1	1.3	1.5	a	1.6	a	1.6	a	1.1	a
400 ACC	8-10 mm	0.0		0.0		0.0		0.1	0.2	b	0.4	bc	1.0	b	1.1		1.3		1.0	0.9	0.5	a	0.3	b	0.3	b	0.1	b
400 ACC	15-20 mm	0.0		0.0		0.0		0.2	0.4	b	0.5	bc	1.5	ab	2.5		1.5		0.8	0.8	0.3	a	0.1	b	0.2	b	0.1	b
10 NAA/400 ACC	8-10/15-20 mm	0.0		0.0		0.0		0.0	0.3	b	0.2	c	1.3	ab	1.6		1.5		0.9	0.9	0.7	a	0.6	b	0.3	b	0.1	b
*P*		–		–		0.1408		0.1343	0.0010	<0.0001			0.0030		0.0970		0.2538	0.1261		0.1015	0.0233	<0.0001			0.0002		0.0017	
Rate of ACC		–		–		NS		NS	NS		NS		NS		L*		NS		NS	NS	Q*	Q***, C**		Q**, C**		Q**, C***		
2018																												
Untreated control		0.0	b	0.3	c	2.4	ab	5.3	4.0	ab	4.1	abc	3.5	abc	2.8	ab	1.7	a	1.4	0.4	0.1		0.2		0.0		0.2	
Hand-thinned control	June drop	0.1	b	0.7	bc	2.4	ab	4.8	4.2	ab	2.4	a-d	2.1	bcd	1.1	b	0.3	a	0.2	0.1	0.1		0.0		0.0		0.1	
1500 CB	8-10 mm	1.0	ab	3.5	a	6.0	a	3.4	1.8	ab	1.4	cd	0.8	cd	0.2	b	0.3	a	0.0	0.1	0.0		0.0		0.0		0.0	
10 NAA	8-10 mm	0.7	ab	2.2	abc	4.9	ab	4.4	3.1	ab	1.7	bcd	1.4	bcd	0.3	b	0.1	a	0.1	0.1	0.0		0.0		0.0		0.1	
1500 CB + 10 NAA	8-10 mm	1.4	a	3.0	ab	1.5	b	1.1	0.3	b	0.2	d	0.1	d	0.0	b	0.0	a	0.0	0.0	0.0		0.0		0.0		0.0	
200 ACC	8-10 mm	0.0	b	0.7	bc	1.7	b	4.1	5.0	a	4.3	ab	4.3	ab	2.2	ab	1.6	a	1.0	0.6	0.6		0.3		0.1		0.3	
300 ACC	8-10 mm	0.1	b	1.1	abc	2.6	ab	2.9	4.4	ab	2.7	a-d	2.5	abcd	2.3	ab	1.2	a	0.5	0.1	0.2		0.2		0.0		0.2	
400 ACC	8-10 mm	0.5	ab	1.3	abc	3.2	ab	3.4	2.5	ab	1.5	bcd	1.5	bcd	1.8	ab	1.8	a	1.2	0.3	0.3		0.3		0.2		0.1	
400 ACC	15-20 mm	0.0	b	0.4	c	1.4	b	3.0	4.1	ab	5.0	a	5.2	a	4.9	a	2.1	a	1.5	0.5	0.2		0.1		0.1		0.1	
10 NAA/400 ACC	8-10/15-20 mm	0.2	b	1.2	abc	3.5	ab	4.3	3.8	ab	2.9	a-d	3.0	abcd	1.5	b	0.3	a	0.4	0.0	0.1		0.0		0.1		0.0	
**Note:** Mean values with the same letter w		0.0010		0.0003		0.0057		0.2567	0.0244	<0.0001		<0.0001			0.0002		0.0170	0.0608		0.0503	0.1953		0.5995		0.3835		0.0650	
Rate of ACC		NS		NS		NS		NS	NS		L**		L*, Q*		NS		NS		NS	NS	NS		NS		NS		NS	

Mean values with the same letter within a given column are not significantly different according to Tukey's HSD test at *P≤* 0.05. NS, *, **, ***, indicates not significant, and significant differences at *P* = 0.05, *P* = 0.01, and *P* =0.001 respectively. L, Q, C refer to linear, quadratic, and cubic relationships.

^a^ Treatment application dates were as follows: 8-10 mm (1 June 2017, 30 May 2018), 15-20 mm (7 June 2017, 8 June 2018).

^b^ Fruit diameter equivalents for each count size: 48 = >98 mm, 56 = 92-95 mm, 72 = 89-92 mm, 80 = 84.5-89 mm, 88 = 83-84.5 mm, 100 = 79-83 mm, 113 = 76-79 mm, 125 = 73-76 mm, 138 = 70-73 mm, 150 = 67-70 mm, 163 = 64-67 mm, 175 = 60-64 mm, 198 = 57-60 mm, 216 = <57.

^c^ The means separation does not reflect all significant comparisons. The following additional pair is significantly different: 1500 CB (8-10 mm), 400 ACC (8-10 mm).

^d^ The means separation does not reflect all significant comparisons. The following additional pair is significantly different: 300 ACC (8-10 mm), 10 NAA (8-10 mm).

Air temperatures one week prior to the application of the first treatments on 1-Jun-2017 were relatively warm ranging from 10 to 25°C with high solar radiation levels four day prior to treatment application (**Figure 1**). Similar conditions prevailed seven days after with high solar radiation. Air temperatures increased daily after the second applications were made on 7-June with high solar radiation levels. Precipitation levels over this period was low, except for 25-May. In 2018, air temperatures were more variable following the first application of treatments on 30-May (**Figure 2**). Air temperatures approached 30°C for the first three days when treatments were made, but declined thereafter with daily highs between 15°C and 20°C, with decrease solar radiation levels. Air temperatures were more moderate following the second application of treatments on 8-June with fluctuating solar radiation levels.

In 2017, ACC increased evolution from fruitlets 24 h after application compared to any of the other treatments (*P* = 0.0059) (**Table 8**). Increasing rates of ACC resulted in an increase in ethylene in a linear fashion. However, ethylene levels in fruitlet from the CB and NAA treatments were similar to the untreated controls, and below 1 nl ethylene g fruit^-1^ hour^-1^. The rise in ethylene was transient and not detected 7 d after application. Ethylene evolution in fruitlets 24 hr and 7 d after the secondary application of 400 mg L^-1^ ACC on 7 June was similar to the untreated control and ranged from undetectable (0) to 0.25 nl ethylene g fruit^-1^ hour^-1^.

**Table 8:** Influence of various rates, combinations and spray timings of Carbaryl (CB), 1-naphthaleneacetic acid (NAA) and 1-aminocyclopropane-1-carboxylic acid (ACC) on the rate of ethylene evolution in dettached fruitlet in 2017 of Honeycrisp/M.9 apples planted in 2012.

	Application timing/fruitlet diameter^a^	Rate of ethylene evolution (nl Ethylene g^-1^ fruit hr^-1^)				
Treatment (mg L^-1^)		24 hours after first spray		7 days after first spray	24 hours after second spray	7 days after second spray
Untreated control		0.72	ab	0.00	0.06	0.00
Hand-thinned control	June drop	–		–	–	–
1500 CB	8-10 mm	0.91	a	0.05	–	–
10 NAA	8-10 mm	0.43	a	0.06	–	–
1500 CB + 10 NAA	8-10 mm	–		–	–	–
200 ACC	8-10 mm	2.17	ab	0.00	–	–
300 ACC	8-10 mm	5.82	a	0.06	–	–
400 ACC	8-10 mm	5.47	ab	0.16	–	–
400 ACC	15-20 mm	–		–	0.25	0.00
10 NAA/400 ACC	8-10/15-20 mm	–		–	–	–
*P*		0.0059		0.1446	NA^c^	–
Rate of ACC		L**		L*		

Mean values with the same letter within a given column are not significantly different according to Tukey's HSD test at *P≤ 0* .05. *, **, indicates significant differences at *P* = 0.05 and *P* = 0.01, respectively. L refers to a linear relationship.

^a^ Treatment application dates were as follows: 8-10 mm (1 June 2017, 30 May 2018), 15-20 mm (7 June 2017, 8 June 2018).

^b^ Not all significant comparisons are shown. The following additional pairs are also significantly different: 400 ACC (8-10 mm), Untreated control.

^c^ could not be calculated by the statistical sofware because the data set was unbalanced.

### 3.3 Experiment 3 – Ambrosia/B.9

Thinning treatments reduced fruit set of Ambrosia in 2017 (*P* = 0.0101), albeit with small differences among treatments (**Table 9**). All thinning treatments, with the exception of the 200 mg L^-1^ rate of ACC, were statistically similar to the HTC. There was no difference in fruit set between the 400 mg L^-1^ rate of ACC applied at 8–10 mm versus 15–20 mm, or between 6-BA followed by 400 mg L^-1^ ACC versus 400 mg L^-1^ ACC applied alone or 6-BA applied alone. Fruit set decreased in a linear fashion with increasing rates of ACC (*P* = 0.05). Thinning treatments in 2018 did not significantly affect fruit set (*P* = 0.1504) of Ambrosia but had a significant effect on the percentage of spurs with zero (*P* = 0.0033), one (*P* = 0.0002) or two fruit (*P* = 0.0031), but not three (*P* = 0.0802) or four fruit (*P* = 0.2239). There were few significant differences in the percentage of spurs with zero fruit according to the means separation. Application of 200 mg L^-1^ ACC tank mixed with 6-BA resulted in the highest percentage of flower spurs with zero fruit, whereas 200 mg L^-1^ ACC applied alone and 400 mg L^-1^ ACC applied at 15–20 mm had the lowest percentage of spurs with zero fruit. The HTC single sprays of 200 mg L^-1^ ACC applied at 8–15 mm, and 400 mg L^-1^ ACC applied at 15–20 mm fruitlet diameter, as well as BA followed by 400 mg L^-1^ ACC had the highest percentage of spurs with one fruit. The HTC had the lowest percentage of spurs with 2 fruit, and only 6-BA applied alone, CB tank mixed with 6-BA, and the tank mixes of ACC and 6-BA were statistically similar to the HTC based on the means separation. Return bloom the spring following treatment application was unaffected by the thinning treatments in either year of the experiment.

**Table 9:** Influence of various rates, combinations and application timings of carbaryl (CB), 6-benzyladenine (6-BA) and 1-aminocyclopropane-1-carboxylic acid (ACC) on fruit set in 2017 and 2018 of Ambrosia/B.9 apples planted in 2005.

	Application timing/fruitlet diameter^a^	Fruit set (no. fruit per 100 flower clusters)		Percentage of flower spurs with indicated number of fruit								Return bloom (% spurs with flowers)^b^	
Treatment (mg L^-1^)				0		1		2		3	4		
2017													
Hand-thinned control	June drop	42.4	b	–		–		–		–	–	1.04	(74.3)
1500 CB	8-10 mm	52.1	ab	–		–		–		–	–	1.10	(79.1)
150 6-BA	8-10 mm	55.2	ab	–		–		–		–	–	1.07	(76.6)
1500 CB + 75 6-BA	8-10 mm	41.2	b	–		–		–		–	–	0.95	(66.3)
200 ACC	8-15 mm	83.4	a	–		–		–		–	–	0.75	(46.7)
300 ACC	8-15 mm	65.4	ab	–		–		–		–	–	0.85	(56.1)
400 ACC	8-15 mm	57.1	ab	–		–		–		–	–	0.86	(57.7)
400 ACC	15-20 mm	67.9	ab	–		–		–		–	–	0.80	(51.0)
75 6-BA/400 ACC	8-10 mm/15-20 mm	55.3	ab	–		–		–		–	–	1.14	(82.4)
200 ACC + 150 6-BA	8-10 mm	68.5	ab	–		–		–		–	–	0.84	(55.0)
300 ACC + 150 6-BA	8-10 mm	71.4	ab	–		–		–		–	–	0.92	(63.6)
*P*		0.0101		–		–		–		–	–	0.1114	
Rate of ACC		L*		–		–		–		–	–	NS	
2018													
Hand-thinned control	June drop	50.3		49.7	ab	50.3	a	0.0	b	0.0	0.0	96	
1500 CB	8-10 mm	55.9		57.1	ab	32.4	b	8.2	a	2.0	0.3	95	
150 6-BA	8-10 mm	71.7		60.1	ab	30.0	b	7.9	ab	1.8	0.0	93	
1500 CB + 75 6-BA	8-10 mm	34.5		69.3	ab	25.6	b	4.7	ab	0.5	0.0	99	
200 ACC	8-15 mm	74.3		46.4	b	37.1	ab	12.7	a	3.3	0.4	87	
300 ACC	8-15 mm	62.8		54.3	ab	31.7	b	11.4	a	1.9	0.7	94	
400 ACC	8-15 mm	63.9		55.1	ab	31.4	b	10.3	a	3.2	0.6	89	
400 ACC	15-20 mm	67.5		46.4	b	41.1	ab	11.1	a	1.4	0.1	88	
75 6-BA/400 ACC	8-10 mm/15-20 mm	67.4		48.4	ab	37.9	ab	11.5	a	2.1	0.0	90	
200 ACC + 150 6-BA	8-10 mm	32.5		71.4	a	23.8	b	4.8	ab	0.2	0.0	97	
300 ACC + 150 6-BA	8-10 mm	41.4		64.8	ab	26.8	b	7.7	ab	0.7	0.0	95	
*P*		0.1504		0.0033		0.0002		0.0031		0.0802	0.2239	0.1175	
Rate of ACC		NS		NS		NS		NS		NS	NS	NS	

Mean values with the same letter within a given column are not significantly different according to Tukey's HSD test at *P≤* 0.05. NS, *, **, **, indicates not significant and significant differences at *P* = 0.05, *P* = 0.01, and *P* = 0.001 respectively. L, Q, C refer to linear, quadratic and cubic relationships.

^a^ Treatment application dates were as follows: 8-10 mm (1 June 2017, 30 May 2018), 15-20 mm (7 June 2017, 5 June 2018).

^b^ Return bloom data from the 2017 trial were transformed using an arcsine square root transformation prior to analyis. Values in brackets are mean values back-transformed to the original scale.

Total fruit yield or percent marketable yield were unaffected by the thinning treatments in either year (**Table 10**). Total number of fruit per tree was significantly affected by the thinning treatments in 2017 and 2018 (*P* = 0.0003 and *P* = 0.0189, respectively) and reflected the degree of thinning achieved by each treatment. In 2017, all thinning treatments resulted in a similar number of fruit as the HTC, except for the 200 mg L^-1^ rate of ACC applied alone or tank mixed with 6-BA. In 2018, there were few significant differences among the treatments as indicated by the means separation and all thinning treatments were statistically similar to the HTC.

**Table 10:** Influence of various rates, combinations and application timings of carbaryl (CB), 6-benzyladenine (6-BA) and 1-aminocyclopropane-1-carboxylic acid (ACC) on tree growth and fruit yield in 2017 and 2018 of Ambrosia/B.9 apples planted in 2005.

Treatment (mg L^-1^)^a^	Application timings/fruitlet diameter	Total fruit yield (kg per tree)	Total number of fruit (no. per tree)	Percent marketable yield (%)		Adjusted mean fruit weight (g)	Crop load (no. fruit per TCSA)^b^		
2017									
Hand-thinned control	June drop	17.2	143	c	100	104		4.3	b
1500 CB	8-10 mm	20.1	182	abc	100	108		6.8	ab
150 6-BA	8-10 mm	25.6	225	abc	100	109		7.3	ab
Tank mix 1500 CB + 75 6-BA	8-10 mm	17.7	167	bc	100	97		6.1	ab
200 ACC	8-15 mm	25.2	295	ab	100	98		9.7	a
300 ACC	8-15 mm	24.1	270	abc	100	99		9.3	ab
400 ACC	8-15 mm	23.6	278	abc	100	91		9.1	ab
400 ACC	15-20 mm	24.3	269	abc	100	98		9.2	ab
75 6-BA followed by 400 ACC	8-10 mm/15-20 mm	18.7	179	abc	100	98		6.4	ab
Tank mix 200 ACC + 150 6-BA	8-10 mm	25.8	301	a	100	96		9.8	a
Tank mix 300 ACC + 150 6-BA	8-10 mm	25.6	279	abc	100	96		8.8	ab
*P*		0.0866	0.0003		–	0.7414		0.0098	
Rate of ACC		NS	NS		–	NS		NS	
2018									
Hand-thinned control	June drop	24.2	128	ab	81	180	ab	2.7	b
1500 CB	8-10 mm	33.0	188	ab	71	173	ab	3.9	ab
150 6-BA	8-10 mm	30.3	170	ab	74	174	ab	3.2	ab
Tank mix 1500 CB + 75 6-BA	8-10 mm	21.7	111	b	85	187	a	2.2	b
200 ACC	8-15 mm	31.5	187	ab	69	170	ab	4.3	ab
300 ACC	8-15 mm	28.3	174	ab	67	151	b	2.8	b
400 ACC	8-15 mm	31.8	195	ab	64	169	ab	3.7	ab
400 ACC	15-20 mm	34.2	228	a	63	162	ab	5.8	a
75 6-BA/400 ACC	8-10 mm/15-20 mm	33.0	192	ab	73	174	ab	4.4	ab
Tank mix 200 ACC + 150 6-BA	8-10 mm	23.3	117	ab	79	188	a	2.2	b
Tank mix 300 ACC + 150 6-BA	8-10 mm	25.9	142	ab	76	178	ab	2.8	b
*P*		0.3254	0.0189		0.0972	0.0542		0.0005	
Rate of ACC		NS	NS		NS	Q*		NS	

Mean values with the same letter within a given column are not significantly different according to Tukey's HSD test at *P≤* 0.05. TCSA, trunk cross-sectional area. NS, *, **, **, indicates not significant and significant differences at *P* = 0.05, *P* = 0.01, and *P* = 0.001 respectively. L, Q, C refer to linear, quadratic and cubic relationships.

^a^ Treatment application dates were as follows: 8-10 mm (1 June 2017, 30 May 2018), 15-20 mm (7 June 2017, 5 June 2018).

^b^ Determined by dividing the total number of fruit harvested with the TCSA measured in fall.

Mean fruit weight, adjusted for crop load, was not significantly affected by the thinning treatments in 2017 (*P* = 0.7414) and was just shy of statistical significance in 2018 (*P* = 0.0542). Although not statistically significant, Tukey’s HSD indicated minor differences among the treatments, and the contrasts indicated a significant curvilinear relationship between mean fruit weight and application rate of ACC.

Thinning treatments had a significant effect on crop load in both 2017 and 2018 (*P* = 0.0098 and *P* = 0.0005, respectively). There were few significant differences among the treatments in either year based on the means separation; however, applications of 200 mg L^-1^ ACC applied alone, or 200 mg L^-1^ ACC tank mixed with 6-BA resulted in significantly higher crop load than the HTC in 2017. In 2018, all thinning treatments were statistically similar to the HTC except for the 400 mg L^-1^ ACC treatment applied at 15–20 mm fruitlet diameter.

Thinning treatments had a significant effect on the size distribution of fruit at harvest in both years of the experiment (**Table 11**). In 2017, only the 198-count size, one of the smallest fruit size categories, was significantly affected by the thinning treatments (*P* = 0.0080). Tank mix applications of 300 mg L^-1^ ACC and 6-BA increased the amount of fruit in this category compared to the HTC. In 2018, the fruit count sizes 138 to 198 were significantly affected by the thinning treatments. Treatments that resulted in a greater degree of thinning, such as the HTC, CB tank mixed with 6-BA and the tank mixes of ACC and 6-BA generally reduced the amount of fruit in these size categories. In contrast, the 400 mg L^-1^ ACC applied at 15–20 mm had significantly more small fruit in the 163, 175, and 198 size categories compared with the HTC.

**Table 11:** Influence of various rates, combinations and application timings of carbaryl (CB), 6-benzyladenine (6-BA) and 1-aminocyclopropane-1-carboxylic acid (ACC) on the weight of fruit per count size in 2017 and 2018 of Ambrosia/B.9 apples planted in 2005.

	Application timing/fruitlet diameter^a^	Amount of fruit per tree in each size category (kg)^b^																			
Treatment (mg L^-1^)		56	64	72	80	88	100	113	125	138		150		163		175		198		216	
2017																					
Hand-thinned control	June drop	0.0	0.0	0.0	0.0	0.5	1.0	2.7	2.7	2.1		1.6		1.2		1.1		2.0	b	2.5	a
1500 CB	8-10 mm	0.0	0.0	0.0	0.0	0.0	0.7	1.9	1.7	3.2		2.0		2.4		1.8		3.1	ab	3.4	a
150 6-BA	8-10 mm	0.0	0.0	0.0	0.0	0.0	0.3	1.1	2.4	3.6		3.6		4.1		3.4		3.8	ab	3.2	a
Tank mix 1500 CB + 75 6-BA	8-10 mm	0.0	0.0	0.0	0.0	0.0	0.1	0.6	1.5	1.9		2.2		2.4		3.1		2.7	ab	3.1	a
200 ACC	8-15 mm	0.0	0.0	0.0	0.0	0.0	0.0	0.3	0.6	0.7		1.0		2.2		2.6		4.7	ab	13.1	a
300 ACC	8-15 mm	0.0	0.0	0.1	0.0	0.3	0.8	1.3	1.6	1.3		1.1		1.7		2.2		3.5	ab	10.2	a
400 ACC	8-15 mm	0.0	0.0	0.0	0.0	0.0	0.0	0.0	0.5	0.9		1.0		1.8		2.1		5.5	ab	11.8	a
400 ACC	15-20 mm	0.0	0.0	0.0	0.0	0.0	0.0	0.3	0.3	1.8		1.8		2.2		2.4		5.1	ab	10.5	a
75 6-BA/400 ACC	8-10 mm/15-20 mm	0.0	0.0	0.0	0.0	0.0	0.0	0.4	1.3	2.2		2.3		2.6		3.2		2.3	b	4.3	a
Tank mix 200 ACC + 150 6-BA	8-10 mm	0.0	0.0	0.0	0.0	0.0	0.0	0.2	0.1	1.2		1.5		2.6		3.5		6.4	ab	11.1	a
Tank mix 300 ACC + 150 6-BA	8-10 mm	0.0	0.0	0.0	0.0	0.0	0.0	0.1	0.2	1.1		1.6		2.7		3.2		6.9	a	9.8	a
*P*		–	–	0.4890	–	0.1220	0.2580	0.1532	0.1463	0.2869		0.1344	0.5193			0.4581		0.0080		0.0044	
Rate of ACC		–	–	Q*	–	NS	Q*	NS	NS	NS		NS		NS		NS		NS		NS	
2018																					
Hand-thinned control	June drop	0.0	0.2	0.5	2.5	3.9	4.3	4.4	3.5	2.5	ab	1.1	ab	0.5	b	0.2	b	0.2	b	0.17	ab
1500 CB	8-10 mm	0.0	0.0	0.4	1.5	3.0	6.4	9.4	5.6	3.3	ab	1.7	ab	1.0	ab	0.5	ab	0.1	b	0.17	ab
150 6-BA	8-10 mm	0.0	0.0	1.4	1.7	3.7	8.2	5.6	4.0	3.0	ab	1.3	ab	0.9	ab	0.3	b	0.2	b	0.05	b
Tank mix 1500 CB + 75 6-BA	8-10 mm	0.1	0.7	1.8	3.3	3.4	5.6	3.9	1.6	0.6	b	0.3	b	0.2	b	0.1	b	0.1	b	0.14	ab
200 ACC	8-15 mm	0.0	0.0	0.0	0.8	2.6	6.7	6.1	6.5	4.2	a	1.8	ab	1.3	ab	0.7	ab	0.6	b	0.07	ab
300 ACC	8-15 mm	0.0	0.2	0.5	1.4	2.5	5.7	6.2	5.7	2.5	ab	1.4	ab	1.3	ab	0.4	ab	0.2	b	0.23	ab
400 ACC	8-15 mm	0.0	0.2	0.9	1.3	3.3	3.7	6.3	6.2	3.6	ab	2.7	ab	1.9	ab	0.8	ab	0.5	b	0.25	ab
400 ACC	15-20 mm	0.0	0.2	0.0	1.1	1.8	4.5	5.9	4.5	5.3	a	3.2	a	3.1	a	1.6	a	1.9	a	0.97	a
75 6-BA/400 ACC	8-10 mm/15-20 mm	0.0	0.0	0.6	3.2	3.2	6.7	6.0	4.7	3.3	ab	2.9	a	1.2	ab	0.4	ab	0.5	b	0.27	ab
Tank mix 200 ACC + 150 6-BA	8-10 mm	0.0	0.5	2.1	3.3	4.0	5.6	4.7	1.7	0.6	b	0.3	b	0.3	b	0.1	b	0.1	b	0.10	ab
Tank mix 300 ACC + 150 6-BA	8-10 mm	0.0	0.2	1.3	2.1	2.1	7.5	5.4	4.0	1.9	ab	0.8	ab	0.3	b	0.1	b	0.1	b	0.16	ab
*P*		0.4539	0.4946	0.1485	0.1604	0.9265	0.3787	0.2488	0.0611	0.0010		0.0007	0.0030			0.0021		0.0002		0.0903	
Rate of ACC		NS	NS	NS	NS	NS	NS	NS	NS	NS		NS		NS		NS		NS		NS	

Mean values with the same letter within a given column are not significantly different according to Tukey's HSD test at *P* ***≤*** 0.05. TCSA, trunk cross-sectional area. NS, * indicates not significant and significant differences at *P* = 0.05. L and C refer to linear and cubic relationships.

^a^ Treatment application dates were as follows: 8-10 mm (1 June 2017, 30 May 2018), 15-20 mm (7 June 2017, 5 June 2018).

^b^ Fruit diameter equivalents for each count size: 56 = 95-98 mm, 64 = 92-95 mm, 72 = 89-92 mm, 80 = 84.5-89 mm, 88 = 83-84.5 mm, 100 = 79-83 mm, 113 = 76-79 mm, 125 = 73-76 mm, 138 = 70-73 mm, 150 = 67-70 mm, 163 = 64-67 mm, 175 = 60-64 mm, 198 = 57-60 mm, 216 = <57 mm.

Air temperatures one week prior to the application of the first treatments on 1-Jun-2017 were relatively warm ranging from 10 to 25°C with high solar radiation levels four day prior to treatment application (**Figure 1**). Similar conditions prevailed seven days after with high solar radiation. Air temperatures increased daily after the second applications were made on 7-June with high solar radiation levels. Precipitation levels over this period was low, except for 25-May. In 2018, air temperatures were more variable following the first application of treatments on 30-May (**Figure 2**). Air temperatures approached 30°C for the first three days when treatments were made, but declined thereafter with daily highs between 15°C and 20°C, with decrease solar radiation levels. Air temperatures were more moderate following the second application of treatments on 8-June with fluctuating solar radiation levels.

### 3.4 Experiment 4 – Ambrosia/M.9

Overall, thinning treatments had a significant effect on fruit set; however, applications of ACC had no effect compared to the untreated control (**Table 12**). Applications of the industry standard tank mix of CB and 6-BA (with and without ACC) resulted in markedly lower fruit set than the other (ACC) treatments. There was no benefit to the addition of a follow-up spray of 400 mg L^-1^ ACC at 18–20 mm fruitlet diameter to the application of CB tank mixed with 6-BA. Overall, there was no difference in the liquid or granular formulations of ACC, perhaps in part because none induced any reduction in fruit set.

**Table 12:** Influence of various rates, formulations (granular or liquid) and combinations of 1-aminocyclopropane-1-carboxylic acid (ACC), Carbaryl (CB), and 6-benzyladenine (6-BA) on fruit set in 2021 of Ambrosia/M.9 apple trees planted in 2012.

	Application timing/fruitlet diameter^a^	Fruit set (no. fruit per 100 flower clusters)		Percentage of flowering spurs with indicated number of fruit										Return bloom (% of spurs with flowers)	
Treatment (mg L^-1^)				0		1		2		3		4	5		
Untreated Control		85.7	a	39.4	b	40.2	a	15.8	a	4.4	ab	0.2	0.0	66.8	d
Hand-thinned control	June Drop	81.5	a	39.6	b	40.8	a	18.2	a	1.5	ab	0.0	0.0	83.1	abc
200 ACC (liquid)	PF, 18-20 mm	90.3	a	35.9	b	42.1	a	18.2	a	3.4	ab	0.3	0.1	64.1	d
400 ACC (liquid)	PF, 18-20 mm	93.0	a	36.9	b	38.5	a	19.7	a	4.6	ab	0.3	0.0	63.5	d
200 ACC (granular)	PF, 18-20 mm	71.6	a	48.0	b	36.1	a	12.8	ab	2.6	ab	0.5	0.0	75.7	bcd
400 ACC (granular)	PF, 18-20 mm	94.5	a	39.4	b	34.7	ab	18.6	a	6.7	a	0.6	0.0	71.1	cd
Tank mix 1000 CB + 75 6-BA	12-15 mm	39.8	b	70.7	a	21.8	c	4.5	bc	3.0	ab	0.0	0.0	91.5	ab
Tank mix 1000 CB + 75 6-BA / 400 ACC (granular)	12-15 mm/18-20 mm	29.1	b	72.9	a	25.0	bc	2.1	c	0.0	b	0.0	0.0	94.8	a
*P*		<0.0001		<0.0001		<0.0001		<0.0001		0.0695		0.6162	0.4478	<0.0001	
Contrasts															
Granular vs Liquid ACC		NS		*		*		NS		NS		NS	NS	*	
200 vs 400 ACC		NS		NS		NS		NS		NS		NS	NS	NS	

Mean values with the same letter within a given column are not significantly different according to Tukey's HSD test at *P≤* 0.05. NS, *, **, *** indicates not significant and significant differences at *P* = 0.05, *P* = 0.01 and *P* = 0.001, respectively.

^a^ Treatment application dates were as follows: Petal fall (PF) (25 May 2021), 12-15 mm (30 May 2021), 18-20 mm (10 June 2021).

The percentage of flowering spurs with zero fruit was higher and the percentages with one or two fruit were lower following applications of CB combined with 6-BA (with and without ACC), compared to the other treatments (**Table 12**). There was no benefit to the addition of a follow-up spray of 400 mg L^-1^ ACC at 18–20 mm fruitlet diameter to CB tank mixed with 6-BA. The granular formulation of ACC increased the percent of spurs with zero fruit (*P* = 0.05) and reduced the percent of spurs with one fruit (*P* = 0.05) compared to the liquid formulation, according to the contrasts. Treatments did not influence the percent of spurs with three fruit; however, there were some minor differences among the treatments. The percentage of spurs with four or five fruit was not significantly affected by the thinning treatments.

Return bloom, recorded the spring following treatment application, was significantly affected by the thinning treatments (*P* < 0.0001) (**Table 12**). The HTC and CB tank mixed with 6-BA (with or without ACC) resulted in higher return bloom than the untreated control or liquid formulation of ACC. Orthogonal contrast indicated that trees sprayed with the granular formulation of ACC had higher return bloom than those sprayed with the liquid formulation (*P* = 0.05) and were statistically similar to the HTC based on Tukey’s HSD.

Total yield from trees sprayed with ACC alone was similar to the untreated control but significantly higher than CB tank mixed with 6-BA treatments (**Table 13**). Increasing the rate of ACC from 200 to 400 mg L^-1^ reduced total yield from an average of 19.5 to 17.6 kg per tree, according to the contrasts (*P* = 0.05). Applications of CB tank mixed with 6-BA, with or without ACC, resulted in the lowest total yields from a numerical perspective. Yields were statistically similar to the HTC but lower than the untreated control according to the means separation.

**Table 13:** Influence of various rates, formulations (granular or liquid) and combinations of 1-aminocyclopropane-1-carboxylic acid (ACC), Carbaryl (CB), and 6-benzyladenine (6-BA) on harvest parameters in 2021 of Ambrosia/M.9 apple planted in 2012.

Treatment (mg L^-1^)	Application timing/fruitlet diameter^a^	Total fruit yield (kg per tree)	Total number of fruit (no. per tree)		Percent marketable fruit (%)		Adjusted mean fruit weight (g)^b^		Crop load (no. fruit per TCSA)^c^		
Untreated Control		18.7	a	145	a	58.4	bc	144	abc	11.5	a
Hand-thinned control	June Drop	14.2	bc	90	b	86.9	a	145	abc	6.7	b
200 ACC (liquid)	PF, 18-20 mm	19.4	a	159	a	39.6	c	139	abc	12.3	a
400 ACC (liquid)	PF, 18-20 mm	17.4	ab	148	a	45.2	c	135	bc	12.4	a
200 ACC (granular)	PF, 18-20 mm	19.7	a	141	a	68.7	ab	151	ab	11.0	a
400 ACC (granular)	PF, 18-20 mm	17.8	ab	137	a	50.9	bc	134	c	9.9	a
Tank mix 1000 CB + 75 6-BA	12-15 mm	11.5	c	65	b	90.7	a	157	a	5.4	b
Tank mix 1000 CB + 75 6-BA / 400 ACC (granular)	12-15 mm/18-20 mm	11.8	c	65	b	88.7	a	157	a	5.1	b
*P*		<0.0001		<0.0001		<0.0001		0.0016		<0.0001	
Contrasts											
Granular vs Liquid ACC		NS		*		**		NS		**	
200 vs 400 ACC		*		NS		NS		**		NS	

Mean values with the same letter within a given column are not significantly different according to Tukey's HSD test at *P≤* 0.05. TCSA, Trunk cross-sectional area. NS, *, **, *** indicates not significant and significant differences at *P* = 0.05, *P* = 0.01, and *P* = 0.001, respectively.

^a^ Treatment application dates were as follows: Petal fall (PF) (25 May 2021); 12-15 mm (30 May 2021), 18-20 mm (10 June 2021).

^b^ Mean fruit weight adjusted using Crop Load as a covariate.

^c^ Determined by dividing the total number of fruit harvested with the TCSA measured in fall.

Total number of fruit and crop load followed a similar trend as total yield. All treatments of ACC applied alone resulted in a similar number of fruit and crop load as the untreated control and a higher fruit number and crop load than the HTC and CB tank mixed with 6-BA treatments. Although not reflected in the means separation, the contrasts indicated a significant reduction in total number of fruit from 153 to 139 fruit per tree (*P* = 0.05) and crop load from 12.4 to 10.5 fruit per TCSA (*P* = 0.01) with the granular versus liquid formulations of ACC.

Treatments that resulted in the greatest reduction in number of fruit per tree also tended to have the highest percent marketable fruit. Percent marketable fruit was highest for the CB tank mixed with 6-BA and HTC treatments. Percent marketable fruit was numerically lowest for the liquid formulations of ACC but was statistically similar to the untreated control. Of the ACC treatments, only the 200 mg L^-1^ rate of granular ACC resulted in a marketable percent similar to the HTC. The contrasts indicated significantly lower marketable percent for liquid ACC than granular ACC (*P* = 0.01).

Thinning treatments also had a significant effect on mean weight of marketable fruit, with those resulting in the greatest degree of thinning tending to have the highest fruit weight. From a numerical perspective, mean fruit weight was highest following application of CB tank mixed with 6-BA, with or without ACC; however, there were no significant differences in mean fruit weight between the chemically thinned treatments and the untreated or HTCs. The orthogonal contrasts indicated a significant reduction in mean fruit weight with applications of 400 versus 200 mg L^-1^ ACC (*P* = 0.01).

Thinning treatments had a marked influence on all fruit count size categories except for the 64, 113 and 216 count sizes (**Table 14**). Treatments that resulted in the greatest degree of thinning tended to have more fruit in the 72 to 100 fruit count categories (fewer, larger fruit per box) and less fruit in the >100 count categories (more, smaller fruit per box). In the 72 to 88 count size categories, CB tank mixed with 6-BA (with and without ACC) significantly increased the weight of fruit compared to the untreated control and was higher than or similar to the HTC. The HTC and CB tank mixed with 6-BA treatments had the highest absolute number of fruit in the 100-count size category but were not significantly different than the untreated control. In the 125 to 198 count size categories, thinning treatments continued to have a highly significant effect overall, although the means separation indicated fewer significant differences among the treatments in the 125, 150, 175 and 198 categories. Applications of ACC alone generally resulted in a higher amount of fruit in the 138 and 163 categories than the CB combined with 6-BA treatments but were statistically similar to the untreated control. In the 175 and 198 count size categories, the liquid formulation of ACC resulted in a greater number of fruit compared to the granular formulation (*P* = 0.05). Increasing the application rate of ACC from 200 to 400 mg L^-1^ decreased the weight of fruit in the 125 category (*P* = 0.05).

**Table 14:** Influence of various rates, formulations (granular or liquid) and combinations of 1-aminocyclopropane-1-carboxylic acid (ACC), Carbaryl (CB), and 6-benzyladenine (6-BA) on the weight of fruit per count size in 2021 of Ambrosia/M.9 apples planted in 2012.

Treatment	Application timing/fruitlet diameter^a^	Amount of fruit per tree in each size category (kg)^b^																						
		64	72		80		88		100		113	125		138		150		163		175		198		216
Untreated Control		0.00	0.00	b	0.00	b	0.16	c	1.56	ab	4.76	3.31	ab	3.22	abc	1.83	ab	1.46	abc	0.77	ab	0.99	ab	0.66
Hand-thinned control	June Drop	0.00	0.00	b	0.06	b	0.66	bc	3.16	a	4.99	3.07	ab	1.22	bc	0.61	b	0.24	bc	0.17	b	0.03	b	0.00
200 ACC (liquid)	PF, 18-20 mm	0.00	0.00	b	0.00	b	0.00	c	0.32	b	2.06	3.89	ab	5.17	a	3.76	a	2.35	ab	0.96	ab	0.84	ab	0.05
400 ACC (liquid)	PF, 18-20 mm	0.00	0.00	b	0.00	b	0.00	c	0.07	b	0.61	2.11	ab	4.40	a	2.87	a	2.98	a	2.10	a	1.66	a	0.64
200 ACC (granular)	PF, 18-20 mm	0.00	0.00	b	0.37	ab	0.29	c	1.38	ab	4.35	4.27	a	3.95	ab	2.22	ab	1.44	abc	0.84	ab	0.51	ab	0.09
400 ACC (granular)	PF, 18-20 mm	0.00	0.00	b	0.00	b	0.06	c	0.70	b	2.40	3.17	ab	5.07	a	3.05	a	2.03	abc	0.94	ab	0.26	ab	0.15
Tank mix 1000 CB + 75 6-BA	12-15 mm	0.05	0.34	a	1.27	a	1.56	ab	3.09	a	2.70	1.28	ab	0.74	c	0.21	b	0.08	c	0.06	b	0.05	b	0.05
Tank mix 1000 CB + 75 6-BA / 400 ACC (granular)	12-15 mm/18-20 mm	0.06	0.29	ab	1.16	a	2.36	a	3.23	a	2.56	0.93	b	0.60	c	0.35	b	0.12	c	0.06	b	0.04	b	0.00
*P*		0.5453	0.0013	<0.0001		<0.0001		<0.0001			0.0524	0.0161	<0.0001			<0.0001		0.0004		0.0007		0.0138		0.1200
Contrasts																								
Granular vs Liquid ACC		NS	NS		NS		NS		NS		NS	NS		NS		NS		NS		*		*		NS
200 vs 400 ACC		NS	NS		NS		NS		NS		NS	*		NS		NS		NS		NS		NS		NS

Mean values with the same letter within a given column are not significantly different according to Tukey's HSD test at *P≤* 0.05. NS, *, **, *** indicates not significant and significant differences at *P* = 0.05, *P* = 0.01, and *P* = 0.001,respectively.

^a^ Treatment application dates were as follows: Petal fall (PF) (25 May 2021), 12-15 mm (30 May 2021), 18-20 mm (10 June 2021).

^b^ Fruit diameter equivalents for each count size: 48 = >98 mm, 56 = 95-98 mm, 64 = 92-95 mm, 72 = 89-92 mm, 80 = 84.5-89 mm, 88 = 83-84.5 mm, 100 = 79-83 mm, 113 = 76-79 mm, 125 = 73-76 mm, 138 = 70-73 mm, 150 = 67-70

mm, 163 = 64-67 mm, 175 = 60-64 mm, 198 = 57-60 mm, 216 = <57 mm.

**Figure 3:**
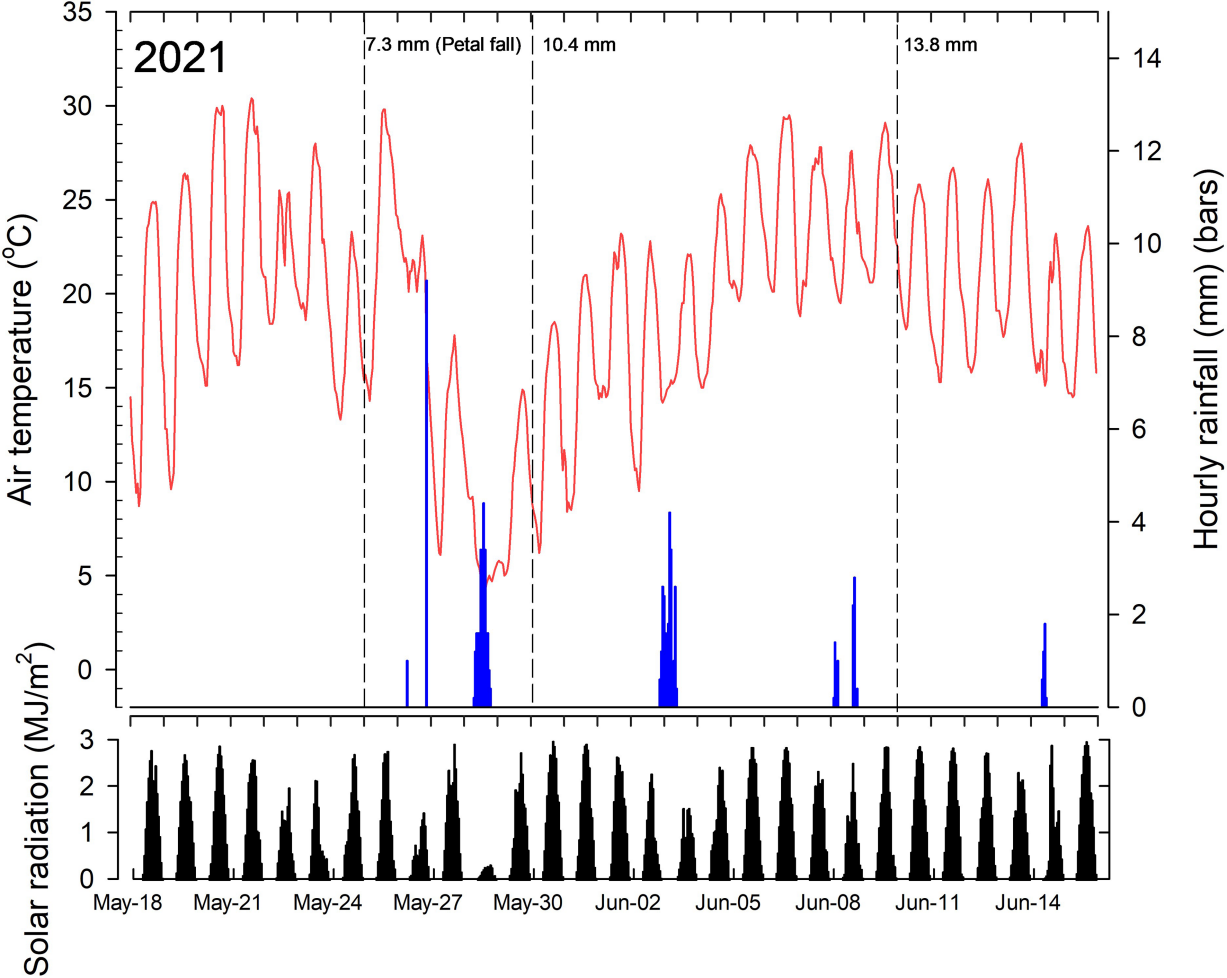
Average hourly air temperatures (red line), total hourly precipitation (blue bars) and solar radiation (black bars) seven days prior to the first application of treatments, and seven days after the last treatments applications to Ambrosia trees in 2021. The dashed vertical line in the top panel indicates the day the treatments were applied and the average diameter of king fruitlets measured on that day. Data recorded at a research weather station located within 500 m of the research orchards.

Consistently warm air temperatures were experienced one week prior to the application of the first treatments on 25-May-2021 with daily highs ranging mid to high 20’s°C with high solar radiation levels five to seven days prior to treatment application followed by decreased levels two and three days prior (**Figure 3**). The day of application, temperatures reached 30°C but then an unstable air mass brought much cooler temperatures with variable solar radiation levels. Air temperatures increased daily after the second applications were made on 30-May with high solar radiation levels. Air temperatures were consistently warm with daily highs in the mid 20°C temperatures with high radiation levels.

### 3.5 Experiment 5 – Crimson Crisp/M.9

In 2022, there were no significant treatment effects of ACC applied at FB or the grower standard CB tank mixed with 6-BA applied at 12–15 mm on fruit set of Crimson Crisp apples (**Table 15**). Orthogonal contrasts also indicated no rate effect of ACC on fruit set. There was also no treatment effect on the percentage of flowering spurs with zero or more fruit per cluster or return bloom the following spring.

**Table 15:** Influence of various rates of 1-aminocyclopropane-1-carboxylic acid (ACC, mg/L) and a tank mix of carbarl and 6-benzyladenine (6-BA) on fruit set in 2022 of 'Crimson Crisp'/ M.9 apple trees.

Treatment	Application timing^a^	Mean fruit set (no fruit/100 flower clusters)	Percentage of flowering spurs with indicated number of fruit							Return bloom (% of spurs with flowers)
			0	1	2	3	4		5	
Untreated Control		82.0	43.0	37.4	15.0	4.0	0.7	a^b^	0.0	0.0
Hand thinned Control	June Drop	74.4	41.4	45.4	12.4	0.7	0.0	b	0.0	77.0
300 ACC	Full bloom	68.2	47.0	42.5	9.2	1.4	0.0	b	0.0	74.3
400 ACC	Full bloom	54.8	56.3	35.0	8.3	0.3	0.1	b	0.0	71.5
500 ACC	Full bloom	66.3	52.4	34.5	10.0	2.6	0.0	b	0.3	75.2
600 ACC	Full bloom	62.9	51.5	36.1	10.4	2.0	0.0	b	0.0	75.8
1500 mg/L CB + 75 mg/L 6-BA	12-15 mm	60.3	50.8	39.3	8.7	1.2	0.0	b	0.0	83.5
*P* value		0.2435	0.1283	0.1297	0.3291	0.2359	0.0394		0.4696	0.3838
Contrasts^c^										
Rate of ACC		ns	ns	ns	ns	ns	ns		ns	ns

^a^ Full bloom sprays applied on 18 May 2022, 12-15 mm sprays applied on 2 June 2022.

^b^ Mean values with the same letter within a given column are not significantly different according to Tukey's HSD test at *P≤* 0.05.

^c^ ns, *, **, ***, indicates not significant, and significant differences at *P* =0.05, *P* =0.01, and *P* =0.001 respectively. L, Q, C refer to linear, quadratic, and cubic.

Thinning treatments applied to Crimson Crisp did influence some yield parameters (**Table 16**); however, none of the ACC treatments ranging from 300 to 600 mg L^-1^ ACC differed from the untreated controls with respect to yield, marketable yield, number of fruit per tree, marketable fruit weight or crop load. Only trees treated with the grower standard CB tank mixed with 6-BA applied at 12–15 mm had similar yields, number of fruit per tree marketable fruit weight, and crop load as the HTC. Furthermore, contrasts indicated no rate effect of ACC on any of the yield parameters. Fruit grading data were collected, but because of lack of treatment difference in size distribution, they are not presented.

**Table 16:** Influence of various rates of 1-aminocyclopropane-1-carboxylic acid (ACC, mg/L) and a tank mix of carbarl and 6-benzyladenine (6-BA) on harvest parameters of 'Crimson Crisp'/ M.9 apple in 2022.

Treatment (mg L-1)	Application timing^a^	Total fruit yield (kg/tree)	Field graded marketable yield (kg/tree)		Total number of fruit (no/tree)	Mean weight of marketable fruit (g)			Crop load (no. fruit /TCSA^d^)		
Untreated Control		17.6	ab	16.7	106	a	170	b	abc	6.4	a
Hand thinned Control	June Drop	13.7	b	13.5	72	b	193	a	ab	4.1	b
300 ACC	Full bloom	17.8	a	17.2	109	a	167	b	bc	6.4	a
400 ACC	Full bloom	18.4	a	17.2	113	a	168	b	abc	6.6	a
500 ACC	Full bloom	17.0	a	16.0	101	ab	173	b	abc	6.4	a
600 ACC	Full bloom	15.5	a	14.9	94	ab	169	b	c	5.7	ab
1500 mg/L CB + 75 mg/L 6-BA	12-15 mm	13.9	b	13.2	73	b	193	a	a	4.5	ab
*P* value		0.0313		0.0876	0.0003		0.0001			0.0018	
Contrasts^e^											
Rate of ACC		ns		ns	ns		ns			ns	

^a^ Full bloom sprays applied on 18 May 2022, 12-15 mm sprays applied on 2 June 2022.

^b^ Mean values with the same letter within a given column are not significantly different according to Tukey's HSD test at *P≤* 0.05.

^c^ Mean weight of marketable fruit adjusted using Crop Load as a covariate.

^d^ Trunk cross-sectional area. Crop load determined by dividing the total number of fruit harvested by the TCSA measured

^e^ ns, *, **, ***, indicates not significant, and significant differences at *P* =0.05, *P* =0.01, and *P* =0.001 respectively. L, Q, C refer to linear, quadratic, and cubic relationships.

**Figure 4:**
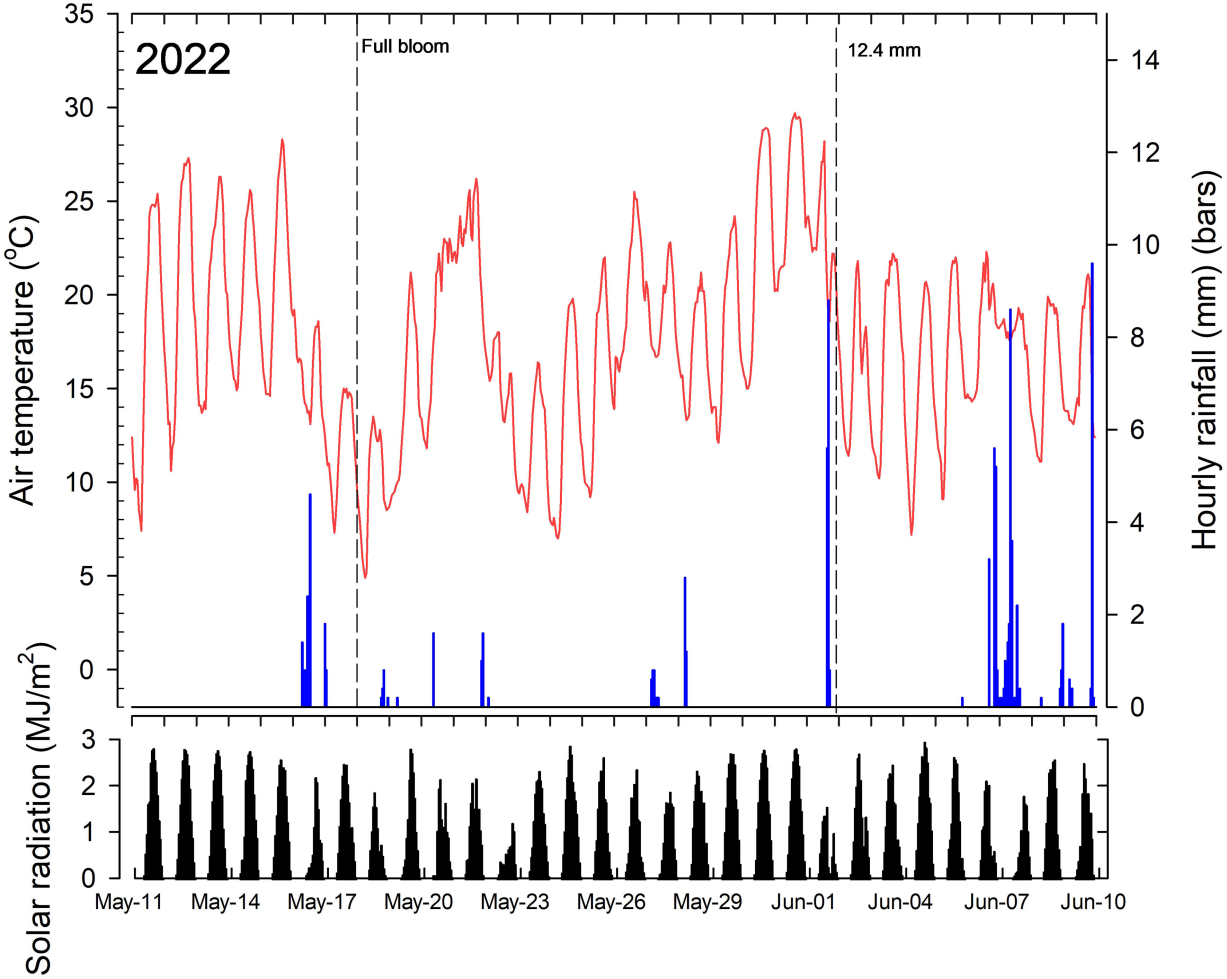
Average hourly air temperatures (red line), total hourly precipitation (blue bars) and solar radiation (black bars) seven days prior to the first application of treatments, and seven days after the last treatments applications to Ambrosia trees in 2022. The dashed vertical line in the top panel indicates the day the treatments were applied and the average diameter of king fruitlets measured on that day. Data recorded at a research weather station located within 500 m of the research orchards.

In 2022, air temperatures were unseasonably warm three to seven days prior to ACC application on 18-May (FB), however cooled to daily highs of 16-18°C two days prior and the day of application (**Figure 4**). Air temperatures then increased to mid 20°C levels followed by cooler temperatures. Solar radiation levels were variable immediately prior to and following applications of ACC at FB. Air temperatures following the application of CB tanked mixed with 6-BA ranged from ~10-20°C for the seven day period following application. Solar radiations were also variable during this period with unsettled weather with significant rain five to seven days following application.

## 4 Discussion

This study investigated various application rates, timings, two formulations, and single and combination sprays of ACC as bloom and post-bloom thinners for Gala, Ambrosia, Honeycrisp, and Crimson Crisp apples over four growing seasons. Over the eight studies, single applications of ACC up to 400 mg L^-1^ for fruitlet sprays and 600 mg L^-1^ for FB sprays failed to reduce fruit set or crop load compared to untreated control trees. Orthogonal contrasts did indicate a significant linear or quadratic reduction in fruit set and crop load with increasing rates of ACC when applied as a post-bloom thinner in three of the six experiments. Trees treated with the grower control treatments of CB tank mixed with 6-BA for Ambrosia and Gala, and CB tank mixed with NAA for Honeycrisp, had inconsistent effects on crop loads when compared with the HTC; and indeed in several years the HTC and untreated control treatments and similar crop loads making it difficult to generalize the best treatment.

These experiments were initiated prior to ACC being registered for commercial use in Canada, when there was a paucity of information in the literature about its thinning efficacy. In 2023, the federal Canadian registrant approved Accede™ SG (formulated as 40% ACC; soluble granular) for thinning apples with a label rate of 200 to 400 mg L^-1^ ACC with an application timing between FB and 25 mm king fruitlet diameter (Pest Management Regulatory Authority, 2023). Furthermore, the manufacturer of Accede™ SG (Valent BioSciences; Valent Canada) indicates it is most active when the king fruitlet diameter is 15–20 mm in diameter (Valent BioSciences, 2025).

The poor thinning response observed with ACC in these studies is surprising given positive results reported by others in the apple literature. Cortens and Cline (2019) found 150 mg L^−1^ ACC was effective for thinning for Gala when applied at 17 mm fruitlet diameter in one of two years, and rates as high as 450 mg L^−1^ resulted in over-thinning. In contrast, Fallahi et al. (2013) indicated under-thinning of Gala at concentrations of 150 mg L^-1^ ACC, whereas 250 and 350 mg L^-1^ ACC were more effective (Fallahi et al., 2013). McArtney and Obermiller (2012) demonstrated that ACC had an extended range of activity between 18- and 27-mm fruitlet diameter when applied at 400 mg L^-1^ ACC to Gale Gala. They also indicated that 200 mg L^-1^ ACC thinned Gold Rush when applied at the earlier timings of 11- and 17-mm fruitlet diameter. Fallahi and McArtney (2022) found 300 mg L^-1^ ACC reduced fruit set of Top Export Fuji and Sun Fuji while 600 mg L^−1^ ACC significantly reduced fruit set in Aztec Fuji. However, crop loads were not reported, and we know there is often a weak relationship between fruit set and crop load (Cline et al., 2022). Petri et al. (2023) demonstrated that 400 and 500 mg L^-1^ ACC applied at PF to Maxi Gala caused excessive thinning, whereas rates of 100 to 300 mg L^-1^ ACC had comparable thinning to their HTC. Moreover, 500 mg L^-1^ caused excessive thinning when applied at 8–10 mm fruitlet diameter. They also observed, as have others, that fruitlets exceeding 25 mm in diameter were less responsive to ACC. Szot et al. (2017) found that combined sprays of 100 to 400 mg L^-1^ ACC with 1.65 kg ha^-1^ metamitron applied at 20-mm fruitlet diameter reduced the number of fruit per tree, but it is unclear how much of this activity was associated with metamitron or ACC because single applications of each at this timing were not evaluated. Beyá-Marshall et al. (2025) found the ACC to be an effective thinner when applied to Gala and Fuji between full bloom and particularly at the 18–25 mm stage; and thinning increase in a dose dependent manner.

However, consistent with the present study, Schupp et al. (2012) found that 100, 300 and 500 mg L^-1^ ACC inadequately thinned Golden Delicious fruitlets when applied at 10-mm fruitlet diameter. Theron et al. (2020) also found inconsistent thinning results with ACC when applied between PF and 20-mm fruitlet diameter to Fuji and Cripps Red apples. Gonzalez Nieto and Robinson (2024) reported that 400 mg L^-1^ ACC applied to Gala at PF did not reduce fruit set or number of fruit per tree when applied alone, but when combined with 150 mg L^-1^ 6-BA had greater efficacy. They also reported in a second study on Honeycrisp that 400 mg L^-1^ ACC reduced the number of fruit per tree when applied at 9- and 11-mm, but not when applied at 17- or 21-mm and that Gala was only responsive to ACC when applied at 19-mm and not earlier when applied at PF, 8-, 11-, or 15-mm.

It is perplexing that the thinning results are so variable with ACC, but perhaps not altogether surprising given that many factors can affect outcomes of chemical thinners (Williams and Edgerton, 1981). In the present study, for each experiment spray volumes were adjusted for differences in canopy size, and sprays were applied dilute and under low wind conditions to ensure thorough cover of the canopy. Further, nozzles and water pressure were consistent among experiments to minimise variability in droplet sizes. Our mixed results with ACC and comparison with other studies may be associated with differences in cultivar, environmental conditions, and methods of application.

Environmental conditions during the fruit set period when thinning sprays are applied in southern Ontario are typically variable because to the influence of the Great Lakes. It is well known that air temperature, wind speed and relative humidity can affect spray drying times, which can lead to variability in plant uptake of ACC. It is also well understood that temperatures can influence tree physiological process, but there are no field or growth chamber studies that have reported on the efficacy of ACC on thinning of apples. Air temperatures are likely to influence endogenous levels of ACC oxidase and base levels of ACC prior to amendment, and consequently the conversion to ethylene. Furthermore, environmental conditions also play a role in the sensitivity of the abscission zone in the fruitlet pedicel. It is beyond the scope of this study to discern pre- and post-environmental conditions on ACC efficacy without fundamental studies which elucidate these factors specifically.

Gonzalez Nieto and Robinson (2024) reported that ACC was more efficacious when applied to trees during a carbohydrate deficit and less effective during a positive carbohydrate balance. Spraying methods can also influence efficacy. For example, several studies used hand sprayers to apply ACC, which tends to result in higher water volumes and active ingredients applied per tree in comparison to air blast sprayer applications. Ultimately, these differences make it challenging to compare study results.

The elevated level of ethylene 24 h after ACC in fruit treated with 300 and 400 mg L^-1^ ACC was not associated with reductions in fruit set or crop load. Enhanced ethylene levels observed in ACC treatments but not in the tank mix treatment of CB and 6-BA supports the mechanism by which exogenous ACC is converted to ethylene, which, in sufficient levels, can lead to fruitlet abscission. Further, the fact that ethylene levels were undetectable in detached fruiting spurs 24 h after ACC was applied at 15–20 mm may explain why there was no fruit abscission associated with this treatment. McArtney (2011) found that ethylene release from detached fruiting spurs increased 1 d after applying 50 mg L^-1^ ACC at full bloom and 10 mm fruitlet diameter, but ACC had a reduced effect on the rate of ethylene release when it was applied after 20 mm fruitlet diameter. Torres et al. (2024) found that abscission response in peach was related to ACC concentration applied at FB, as well as to ethylene emissions, which also peaked 1 d after application. An increase in abscission is consistent with the purported model for abscising apple fruitlets, which maintains that when fruitlets reach a given threshold of ethylene, abscission cues upregulate ethylene genes and trigger fruit abscission (Eccher et al., 2015). In the present study, it’s plausible that increased endogenous ethylene level were insufficient to trigger fruit abscission at the rates of ACC use. Alternatively, maybe ethylene alone is not enough to trigger fruitlet abscission or possibly it needs to be combined with another physiological trigger such as a carbohydrate deficit (Lordan et al., 2019; Robinson et al., 2010). Also, perhaps genetic differences or environmental cues limited endogenous levels of the ACC oxidase enzyme and its conversion ethylene (Binnie et al., 2007).

Early fruit thinning improves flower initiation and hence return bloom for the following season (Dennis, 2003). Thinning treatments influenced return bloom in three of the eight experiments. Treatments had no effect on return bloom of Gala trees in 2017 and 2018 most likely because crop loads were very light and not inhibiting flower initiation the years the treatments were applied. Return bloom was improved in Honeycrisp (2017, 2018) and Ambrosia (2021) trees, which generally corresponded with treatment efficacy in reducing crop load the year prior. There appeared to be little direct effect of ACC on enhancing flower bud initiation independent of thinning; this is currently an area of active research based on the premise that increased endogenous ethylene has been shown to increase flowering (Gonzalez Nieto and Robinson, 2024).

Notwithstanding the importance of these findings, there were some limitations beyond our experimental control. First, in 2017 and 2018, final crop loads of untreated trees that were also not hand thinned were low, with 2.1 and 2.8 fruit/cm^2^ TCSA, well below the optimum of 5–6 fruit/cm^2^ required for a commercial crop for these trees (Cline, 2009a, 2009b). There is evidence that trees with lighter crop loads are typically more difficult to thin than those that set a heavy number of fruit, and this could explain the lack of treatment response in these years. Despite this, untreated Honeycrisp trees with slightly higher crop loads in 2018 treated with 4.8 fruit/cm^2^ TCSA responded well to CB and NAA when applied alone or tank mixed. Recent research by Culeman et al (2025) is addressing some fundamental ACC research on apples.

### 4.1 Summary and future research

Overall, ACC was at most a mild thinner in the present studies, and often was an ineffective thinner for Ambrosia, Gala, Honeycrisp and Crimson Crisp even in years with high fruit set and crop loads on untreated trees. In many instances CB, NAA, 6BA or ACC used in combination or sequence with these treatments reduced the target crop load with the ideal range of 5–7 fruit cm^-2^ TCSA. However, ACC treatments alone even at 400 mg L^-1^ failed to achieve target crop loads and fruit size for commercial production for the growing region. Contrary to our hypotheses, ACC does not appear to be a sufficient substitute for CB. Future research should investigate higher rates of ACC efficacy, perhaps using ACC in combination with other thinning agents, and aim to understand the reasons for its inconsistent thinning response, particularly the influence of environmental conditions on ACC activity. It is common to have inconsistent thinning responses with hormonal plant growth regulators as many factors influence the thinning response. These include environmental conditions on leaf and fruit cuticle development, product uptake, product drying times, solar radiation on plant carbohydrate balance to name a few. It will be difficult to replace carbaryl with an equally effective and consistent hormonal based thinner. Greater understanding of ACC-induced fruit ethylene evolution as well as environmental conditions at and following ACC application on tree and fruit physiology are two avenues worthy of consideration.

## Data Availability

The original contributions presented in the study are included in the article/supplementary material. Further inquiries can be directed to the corresponding author.

## References

[B1] Beyá-Marshall V. Verdugo A. Fichet T. Reginato G. (2025). The efficacy of 1-aminocyclopropane-1-carboxylic acid (ACC) in thinning apples under Chilean conditions. Eur. J. Agron. 168, 127606. doi: 10.1016/j.eja.2025.127606. PMID: 41868780

[B2] Binnie J. E. Tustin S. McManus M. T. (2007). “ Characterisation of expression of the ACC oxidase gene family of apple (Malus domestica),” in Advances in plant ethylene research. Eds. Ramina A. Chang C. Giovannoni J. Klee H. Perata P. Woltering E. ( Springer, Dordrecht). doi: 10.1007/978-1-4020-6014-4_8, PMID:

[B3] Cline J. A. (2009a). Commercial production of Honeycrisp apples in Ontario. Available online at: https://www.ontario.ca/page/commercial-production-honeycrisptm-apples-ontario (Accessed January 12, 2026).

[B4] Cline J. A. (2009b). Commercial production of Ambrosia apples in Ontario. Available online at: https://www.ontario.ca/page/commercial-production-ambrosiatm-apples-ontario (Accessed January 12, 2026).

[B5] Cline J. A. Bakker C. J. Beneff A. (2020). Thinning response of ‘Redhaven’ peaches to 1-aminocyclopropane carboxylic acid (1-ACC). Can. J. Plant Sci. 101, 17–29. doi: 10.1139/cjps-2020-0018. PMID: 36563491

[B6] Cline J. A. Bakker C. J. Beneff A. (2022). Multi-year investigation on the rate, timing, and use of surfactant for thinning apples with post-bloom applications of metamitron. Can. J. Plant Sci. 102, 628–655. doi: 10.1139/cjps-2021-0206. PMID: 36563491

[B7] Cline J. A. Carter K. Gunter A. Bakker C. Green A. C. (2017). Response of Bosc and Cold Snap™ pears to thinning with NAA, 6-BA, ACC, and s-ABA. Can. J. Plant Sci. 98, 830–843. doi: 10.1139/cjps-2017-0258. PMID: 36563491

[B8] Cortens M. H. Cline J. A. (2019). Effects of the bioregulators ACC, 6-BA, ABA, and NAA as thinning agents on Gala apples. Can. J. Plant Sci. 100, 185–201. doi: 10.1139/CJPS-2019-0014. PMID: 36563491

[B9] Culemann E. Winkler A. Brinkmann T. Knoche M. (2025). 1-aminocyclopropane-1-carboxylic acid (ACC) increases ethylene evolution and fruit abscission in developing apple spurs (Malus x domestica Borkh.). J. Plant Growth Regul. 44 (9), 5290–5301. doi: 10.1007/s00344-025-11759-8, PMID: 30311153

[B10] Dennis F. (2003). “ Flowering, pollination and fruit set and development,” in Apples: botany, production and uses. Eds. Ferree D. C. Warrington I. J. ( CAB Publishing, CAB International, Wallingford, UK), 153–166.

[B11] Eccher G. Begheldo M. Boschetti A. Ruperti B. Botton A. (2015). Roles of ethylene production and ethylene receptor expression in regulating apple fruitlet abscission. Plant Physiol. 169, 125–137. doi: 10.1104/pp.15.00358. PMID: 25888617 PMC4577387

[B12] Fallahi E. Kiester M. J. Fallahi B. Greene D. W. (2013). Influence of potentially new post-bloom thinners on apple fruit thinning. Acta Hortic. 1042, 183–188. doi: 10.17660/actahortic.2014.1042.22

[B13] Fallahi E. McArtney S. J. (2022). Impacts of 1-aminocyclopropane-1-carboxylic acid as a late post-bloom thinner on fruit set, yield, and fruit quality in “Gala” and “Fuji” apples. Am. J. Plant Sci. 13, 481–493. doi: 10.4236/ajps.2022.134031

[B14] Gonzalez Nieto L. Robinson T. (2024). Fruit thinning and flower induction with 1-aminocyclopropane-1-carboxylic acid (ACC Accede). Fruit Q. 32, 27–30. doi: 10.17660/actahortic.2025.1443.9

[B15] Health Canada (2016). Re-evaluation Decision RVD2016-02-carbaryl. Available online at: https://www.Canada.ca/content/dam/hc-sc/migration/hc-sc/cps-spc/alt_formats/pdf/pubs/pest/_decisions/rvd2016-02/rvd2016-02-eng.pdf (Accessed January 12, 2026).

[B16] Health Canada (2024). Changes to the registration of carbaryl pesticides. Available online at: https://www.Canada.ca/en/health-Canada/services/publications/product-safety/changes-registration-carbaryl-pesticides.html (Accessed January 12, 2026).

[B17] Hohner B. Presant T. (1989). Soils of the horticultural experiment station simcoe Vol. Publ. 89-3 (Guelph, Ontario: Ont. Inst. Pedol).

[B18] Lakso A. N. (2011). Early fruit growth and drop-the role of carbon balance in the apple tree. Acta Hortic. 903, 733–742. doi: 10.17660/actahortic.2011.903.102

[B19] Lordan J. Reginato G. H. Lakso A. N. Francescatto P. Robinson T. L. (2019). Natural fruitlet abscission as related to apple tree carbon balance estimated with the MaluSim model. Sc. Hortic. 247, 296–309. doi: 10.1016/j.scienta.2018.11.049. PMID: 41868780

[B20] McArtney S. J. (2002). Ethylene evolution from detached apple spurs in response to chemical thinners. HortScience 37, 662–665. doi: 10.21273/HORTSCI.37.4.662

[B21] McArtney S. J. (2011). Effects of 1-aminocyclopropane carboxylic acid on the rate of ethylene release from detached fruiting spurs and on fruit abscission in apple. J. Hortic. Sci. Biotech. 86, 640–644. doi: 10.1080/14620316.2011.11512816. PMID: 41858497

[B22] McArtney S. J. Obermiller J. D. (2012). Use of 1-aminocyclopropane carboxylic acid and metamitron for delayed thinning of apple fruit. HortScience 47, 1612–1616. doi: 10.21273/HORTSCI.47.11.1612

[B23] McArtney S. Petracek P. Francescatto P. Forney K. (2022). Thinning apples and stone fruit with the naturally occurring compound 1-aminocyclopropane carboxylic acid. Acta Hortic. 1344, 23–28. doi: 10.17660/ActaHortic.2022.1344.5

[B24] Ontario Apple Growers (2024). Annual report of the ontario apple growers. Available online at: https://www.onapples.com/uploads/images/files/OAG%20Annual%20Report%202024%20FINAL.pdf (Accessed January 12, 2026).

[B25] Ontario Ministry of Agriculture, Food and Agribusiness (2024). Thinning of tree fruit. Available online at: https://www.ontario.ca/page/thinning-tree-fruit#section-4 (Accessed January 12, 2026).

[B26] Ontario Ministry of Agriculture, Food, and Rural Affairs (2018). Fruit production recommendations 2016-2017 (Toronto, Ontario: Queens Printer).

[B27] Pest Management Regulatory Authority (2023). Accede™ SG plant growth regulator – registration no: 34861 pest control products act. Available online at: https://pr-rp.hc-sc.gc.ca/ls-re/index-eng.php (Accessed January 12, 2026).

[B28] Petri J. L. Sezerino A. A. Couto M. (2023). The efficiency of ACC in the fruit thinning of Maxi Gala apple applied in four stages and five concentrations. Acta Hortic. 1366, 261–268. doi: 10.17660/actahortic.2023.1366.30

[B29] Presant E. W. Acton C. J. (1984). The soils of the regional municipality of Haldimand-Norfolk. Report No. 57 of the Ontario Institute of Pedology. Volume 1 (Guelph, ON Canada: Ontario Ministry of Agriculture and Food). Available online at: http://sis.agr.gc.ca/cansis/publications/surveys/on/on57/on57-v1_report.pdf (Accessed January 12, 2026).

[B30] Robinson T. L. Lakso A. N. Hoying S. A. (2010). Advances in predicting chemical thinner response of apple using a MaluSim carbon balance model. Acta Hortic. 932, 223–229. doi: 10.17660/actahortic.2012.932.32

[B31] Schupp J. R. Kon T. M. Winzeler H. E. (2012). 1-aminocyclopropane carboxylic acid shows promise as a chemical thinner for apple. HortScience 47, 1308–1311. doi: 10.21273/HORTSCI.47.9.1308

[B32] Solomakhin A. A. Blanke M. M. (2010). Mechanical flower thinning improves the fruit quality of apples. J. Sci. Food. Agric. 90, 735–741. doi: 10.1002/jsfa.3875. PMID: 20355106

[B33] Sutton T. B. Unrath C. R. (1988). Evaluation of the tree-row-volume model for full-season pesticide application on apples. Plant Dis. 72, 629–632. doi: 10.1094/PD-72-0629. PMID: 40211709

[B34] Szot I. Lipa T. Krawiec P. Basak A. (2017). The estimation of effectiveness of ATS, metamitron, 6-BA and ACC in flower and fruitlet thinning of 'Jonagold Red Prince' apple trees. Act. Hortic. 1221, 39–44. doi: 10.17660/actahortic.2018.1221.6

[B35] Theron K. I. Lötze G. F. A. Scholtz D. A. Reynolds J. S. (2020). The efficacy of 1-aminocyclopropane-1-carboxylic acid (ACC) as a chemical thinner on apples. Acta Hortic. 1341, 15–24. doi: 10.17660/actahortic.2022.1341.3

[B36] Torres E. Caimel D. Asín L. (2024). Responses of ethylene emission, abscission, and fruit quality to the application of ACC as a chemical thinner in ‘Flatbeauti’ peach. J. Plant Growth Regul. 43, 4171–4184. doi: 10.1007/s00344-024-11382-z. PMID: 41868966

[B37] Valent BioSciences (2025). Accede SG plant growth regulator soluble granule. Available online at: https://www.valent.com/products/accede-sg#label (Accessed January 12, 2026).

[B38] Wallis A. Schwallier P. Irish-Brown A. (2022). Thinning strategies for 2022. Available online at: https://www.canr.msu.edu/news/honeycrisp-crop-management (Accessed January 12, 2026).

[B39] Williams M. W. Edgerton L. J. (1981). “ Fruit thinning of apples and pears with chemicals,” in Agriculture information bulletin no. 289 ( U.S. Department of Agriculture, Science and Education Administration, Washington, DC).

[B40] Zanrosso A. Dias A. F. Baldissera S. Fagherazzi A. F. Rusin C. Rufato D. P. . (2025). Efficacy of ACC (1-aminocyclopropane-1-carboxylic acid) as a late chemical thinner for ‘Fuji Mishima’ apple. Fruit Crops Sci. J. 1, e-089. doi: 10.1590/3085-89092025089. PMID: 41821979

